# A PAX6-regulated receptor tyrosine kinase pairs with a pseudokinase to activate immune defense upon oomycete recognition in *Caenorhabditis elegans*

**DOI:** 10.1073/pnas.2300587120

**Published:** 2023-09-19

**Authors:** Florence Drury, Manish Grover, Mark Hintze, Jonathan Saunders, Michael K. Fasseas, Charis Constantinou, Michalis Barkoulas

**Affiliations:** ^a^Department of Life Sciences, Imperial College, London SW7 2AZ, United Kingdom

**Keywords:** oomycete, *C. elegans*, innate immunity, receptor tyrosine kinase, PAX6

## Abstract

Oomycetes were recently discovered as natural pathogens of *Caenorhabditis elegans,* and pathogen recognition alone was shown to be sufficient to activate a protective transcriptional program characterized by the expression of multiple *chitinase-like* (*chil*) genes. However, the molecular mechanisms underlying oomycete recognition in animals remain fully unknown. We performed here a forward genetic screen to uncover regulators of *chil* gene induction and found several independent loss-of-function alleles of *old-1* and *flor-1,* which encode receptor tyrosine kinases belonging to the *C. elegans*-specific KIN-16 family. We report that OLD-1 and FLOR-1 are both necessary for mounting the immune response and act in the epidermis. FLOR-1 is a pseudokinase that acts downstream of the active kinase OLD-1 and regulates OLD-1 levels at the plasma membrane. Interestingly, the *old-1* locus is adjacent to the *chil* genes in the *C. elegans* genome, thereby revealing a genetic cluster important for oomycete resistance. Furthermore, we demonstrate that *old-1* expression at the anterior side of the epidermis is regulated by the VAB-3/PAX6 transcription factor, well known for its role in visual system development in other animals. Taken together, our study reveals both conserved and species-specific factors shaping the activation and spatial characteristics of the immune response to oomycete recognition.

## Significance

The nematode *Caenorhabditis elegans* can recognize natural oomycete pathogens and activate a protective transcriptional program called the oomycete recognition response. We discovered that two receptor tyrosine kinases (OLD-1 and FLOR-1) from the previously uncharacterized and *C. elegans*-specific KIN-16 family are required to mediate this response. In addition, we found that *old-1* expression is regulated by the worm ortholog of PAX6 (VAB-3), which is an evolutionarily conserved transcription factor widely known for its role in the development of the visual system. OLD-1, FLOR-1, and VAB-3 are the first set of regulators identified for oomycete recognition in animals and unravel the interplay of species-specific and conserved factors necessary for defense against pathogenic oomycetes.

Immune responses to infections are vital for the survival of all organisms. *Caenorhabditis elegans* is a small, free-living nematode that is used as a model to understand the molecular pathways employed by animal hosts to fight infection. *C. elegans* encounters a plethora of pathogens in its natural environment, and sampling of wild isolates over the last years has allowed new natural host–pathogen interaction systems to be established for investigation in the laboratory (1). Immune responses to naturally occurring infections have likely coevolved alongside the pathogens and have been studied in the context of bacterial (2, 3), viral (4), fungal (5, 6), and fungal-like microsporidia infections (7). We previously reported a new natural infection of *C. elegans* by the oomycete *Myzocytiopsis humicola* (8). Oomycetes are eukaryotic pathogens that cause lethal infection in both plants (9) and animals (10). For example, human oomycete infection is caused by the oomycete *Pythium insidiosum* and represents an emerging tropical infection with a high mortality rate due to lack of awareness and suitable noninvasive treatments (11). Therefore, *C. elegans* provides a useful model host to increase our knowledge on oomycete infections at a whole-animal level.

*M. humicola* rapidly kills *C. elegans* by attaching and penetrating through its cuticle and developing first into hyphae and then sporangia inside the nematode body cavity before the release of zoospores that go on to infect new animals (8). Interestingly, *C. elegans* can detect *M. humicola* in the environment using chemosensory neurons (12). This detection likely triggers cross-tissue communication from the neurons to the epidermis where *chitinase-like* (*chil*) genes are induced and offer protection against infection through modification of the outer barrier, the cuticle (8). A similar kind of neuron-to-epidermis communication also exists for immune signaling against fungal infection in *C. elegans*, where neuronal expression of *dbl-1* regulates expression of *cnc* antimicrobial peptides in the epidermis (13). The induction of *chil* genes can be elicited by an innocuous oomycete extract produced by washing off plates containing infected animals, followed by filter sterilization and autoclaving. By exposing animals to such extracts, an oomycete recognition response (ORR) has been described, which includes genes induced in common upon extract treatment and oomycete infection (12). Animal recognition of oomycete pathogens is likely to be a general feature, for example, it has been previously shown that cell wall components from the oomycete *Saprolegnia parasitica* and glucan extract from *P. insidiosum* activate Th2-like response (14) and a specific Th1/Th17 response (15, 16) in Atlantic salmon and BALB/c mice, respectively. Furthermore, the proinflammatory response in Atlantic salmon was found to extend to multiple tissues beyond gills like head kidney cells as well as the mucosal tissue (14). However, the underlying mechanisms of oomycete recognition and the exact machinery required for triggering these responses remain fully unknown. Identifying regulators of ORR in *C. elegans* can offer insights into systemic animal responses to oomycete infection.

In this study, we have started dissecting the underlying signaling pathway involved in oomycete recognition by identifying the first positive regulators of the ORR. We show that overexpression longevity determinant (OLD-1) and full loss of oomycete recognition (FLOR-1), which are predicted receptor tyrosine kinases from the KIN-16 family in *C. elegans,* are both required in the epidermis for mounting the ORR. We establish OLD-1 as an active kinase driving the response, while FLOR-1 as a pseudokinase that regulates both OLD-1 levels at the membrane and the downstream signaling. Furthermore, we identify the transcription factor VAB-3, which is the *C. elegans* homolog of PAX6, as being necessary for the activation of the ORR through influencing the expression of *old-1.* This work therefore describes a unique kinase-pseudokinase pair that is spatially regulated by the activity of a conserved developmental factor to allow pathogen-specific immune signaling in *C. elegans*.

## Results

### *old-1* and *flor-1* Are Required for Oomycete Recognition in *C. elegans*.

**Fig. 1.**
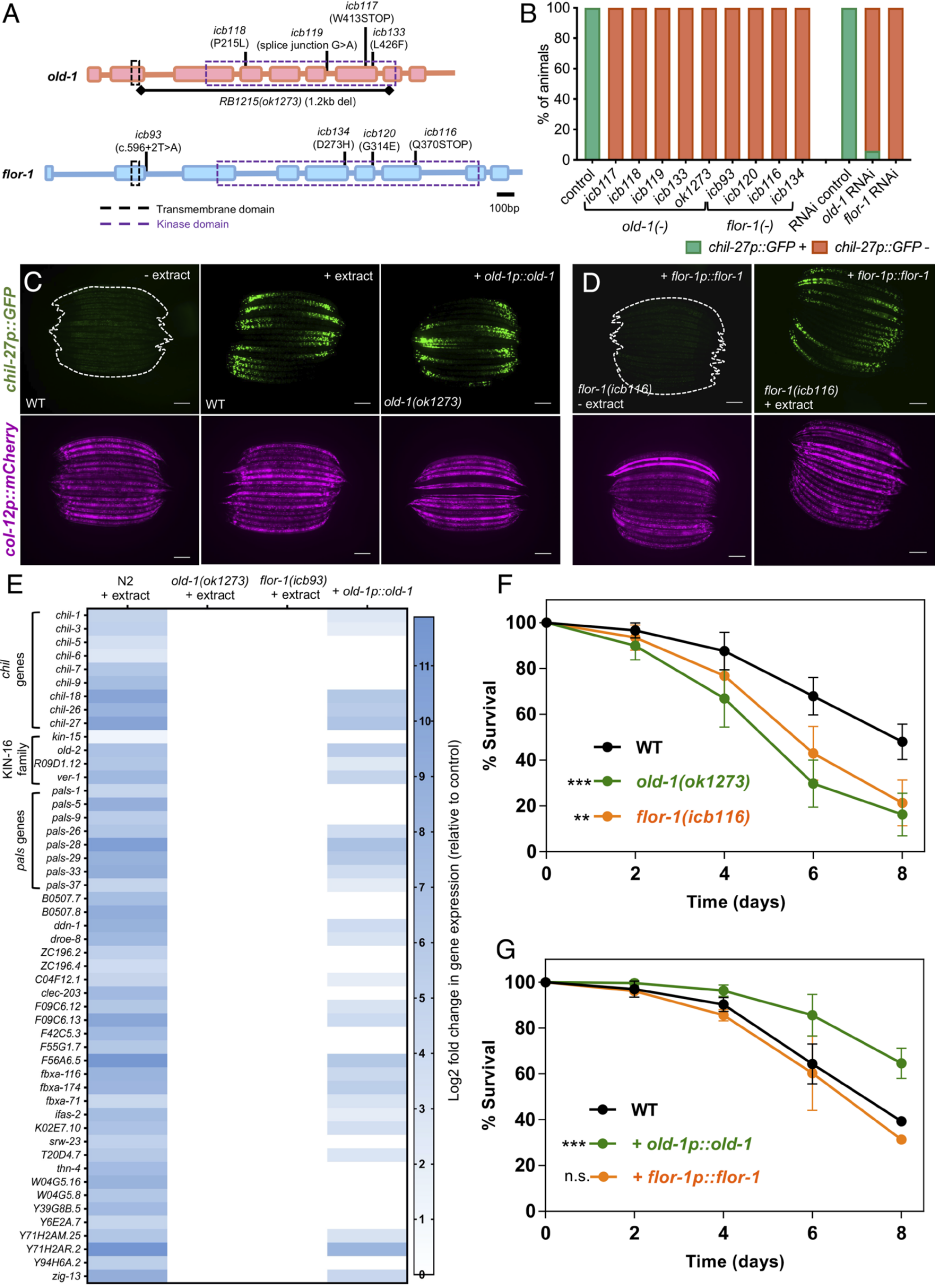
*old-1* and *flor-1* are required for the oomycete recognition response in *C. elegans.* (*A*) Gene structure of *old-1* and *flor-1* and location of mutations recovered. (*B*) Mutants carrying these mutations show a 100% loss of response to *M. humicola* extract. RNAi-mediated knock-down of *old-1* or *flor-1* phenocopies the reduction in response to *M. humicola* extract (chi-square test for each sample, ***P < 0.001, n > 50 animals). (*C*) Overexpression of *old-1(icbEx308)* in *old-1(ok1273)* causes constitutive activation of *chil-27p::GFP* mimicking extract treatment. (*D*) Overexpression of *flor-1(icbEx435)* in *flor-1(icb116)* mutant does not cause constitutive activation of *chil-27p::GFP* but rescues response to extract treatment. For panels (*C* and *D*), the presence of *col-12p::mCherry* represents a constitutive marker that is expressed in adults, n > 100 and Scale bar is 100 µm. (*E*) Heatmap showing the induction of the 50 most consistently upregulated ORR genes. Note that none of these ORR genes are transcriptionally up-regulated in *old-1(ok1273)* and *flor-1(icb93)* mutants whereas around two-thirds are induced upon *old-1* overexpression (*icbEx308*, compared to nontransgenic animals from the same population as control). Significant (*q* value < 0.1, *P* value < 0.01) log2 fold changes are shown in blue, while white represents nonsignificance. (*F*) Loss-of-function of *old-1* or *flor-1* increase susceptibility to *M. humicola* infection. (*G*) Overexpression of *old-1p::old-1(icbEx358)* makes animals less susceptible to *M. humicola* infection, while animals overexpressing *flor-1* behave similar to WT animals. WT control used for this experiment also carries the hygromycin resistance (*icbEx360*). For panels (*F* and *G*), n = 80 to 90 animals per condition, representative graph of three repeats is shown and stars indicate significance with a Log rank test as follows: **P < 0.01, ***P < 0.001, ****P < 0.0001.

To dissect the machinery involved in oomycete recognition, we performed a forward genetic screen using the induction of the *chil-27p::GFP* reporter as a read-out for pathogen detection. Animals carrying this marker were mutagenized and their F2 progeny was screened for loss of GFP expression upon exposure to *M. humicola* extract. Around 60,000 haploid genomes were screened and multiple independent mutations were recovered in two genes, *old-1* and *flor-1*, (Fig. 1*A*) that led to complete loss of *chil-27p::GFP* induction upon extract treatment (Fig. 1*B*). Loss of *chil-27p::GFP* induction could be phenocopied by *old-1* and *flor-1* RNAi and was also observed in a background carrying a genetic deletion of *old-1(ok1273),* suggesting that the isolated mutations from the screen represent loss-of-function alleles (Fig. 1*B*). OLD-1 and FLOR-1 are part of the KIN-16 family of receptor tyrosine kinase (RTKs) family, which is *C. elegans-*specific and its members are little characterized (17–19).

Overexpression of RTKs can lead to ligand-independent activation of downstream signaling events (20, 21); therefore, we tested the effect of overexpressing *old-1* and *flor-1* in the respective mutants. While overexpression of *old-1p::old-1* in *old-1(ok1273)* mutants resulted in constitutive activation of *chil-27p::GFP*, overexpression of *flor-1p::flor-1* in *flor-1(icb116)* restored the response to *M. humicola* extract, yet did not lead to constitutive GFP fluorescence (Fig. 1 *C* and *D*). Consistent with this result, we found significantly reduced induction of *chil-27* by qPCR upon *flor-1* overexpression in comparison to *old-1* overexpression (*SI Appendix*, Fig. S1).

To determine whether loss of *chil-27p::GFP* induction in *old-1(-)* and *flor-1(-)* mutants upon extract treatment is representative of a broader loss of induction of ORR genes, we performed RNA-sequencing (*SI Appendix*, Dataset S1). The transcriptomic results of wild-type, extract-treated animals were first compared to previously published ORR datasets (12) obtained using comparable conditions (L4 stage animals and 4 h post extract treatment). We found 50 genes consistently upregulated in response to *M. humicola* extract in multiple independent experiments (Fig. 1*E*). These genes include members of the *chil* gene family, members of the KIN-16 family to which *old-1* and *flor-1* belong, and *pals* genes, which are also part of the intracellular pathogen response (IPR) induced upon infection with microsporidia or the Orsay virus in *C. elegans* (22). We found that loss of *old-1* or *flor-1* function impaired the induction of all 50 genes upon extract treatment (Fig. 1*E* and *SI Appendix*, Dataset S1). Additionally, more than 50% of these genes were found to be upregulated upon overexpression of *old-1* under its own promoter, suggesting that overexpression of *old-1* can largely mimic the induction of ORR upon extract treatment (Fig. 1*E* and *SI Appendix*, Dataset S1). Consequently, *old-1(-)* and *flor-1(-)* mutants survived less upon infection by *M. humicola* than wild-type animals (Fig. 1*F*). Conversely, constitutive activation of ORR through overexpression of *old-1p::old-1* caused increased survival to *M. humicola* infection, while animals overexpressing *flor-1p::flor-1* showed similar susceptibility to wild-type animals (Fig. 1*G*). These results establish OLD-1 and FLOR-1 as key players for the activation of oomycete recognition response in *C. elegans*.

### OLD-1 and FLOR-1 Are Required in the Epidermis for the Induction of ORR.

**Fig. 2.**
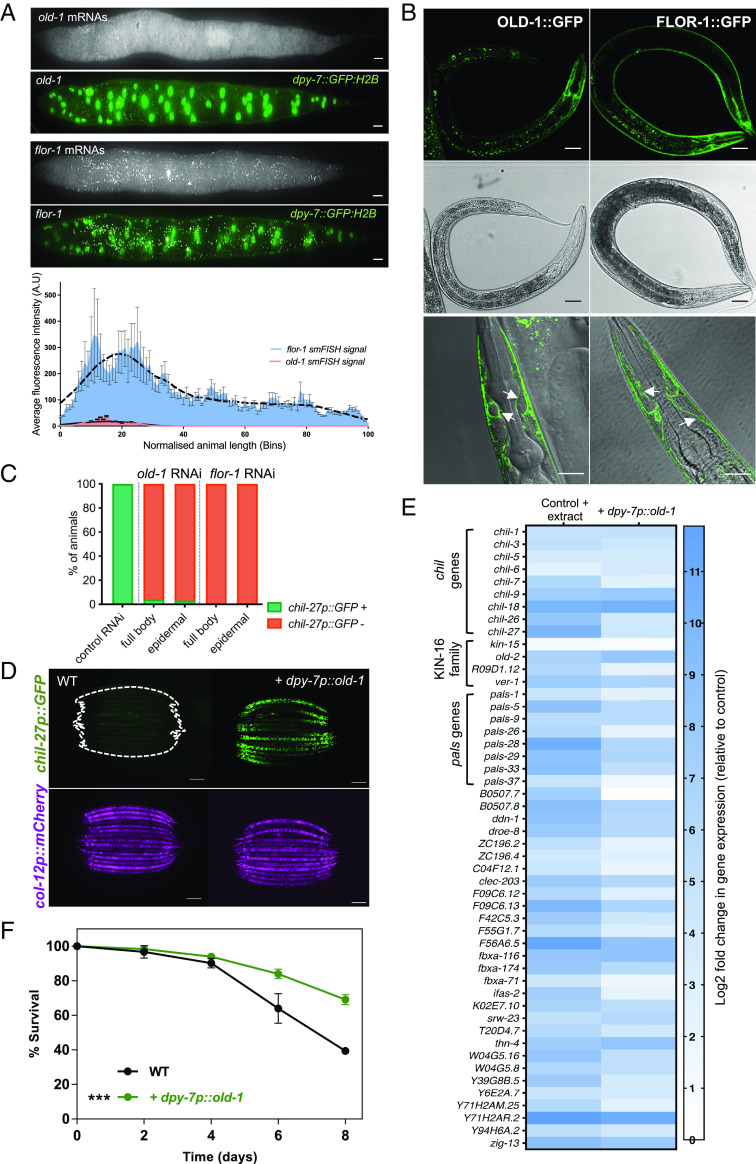
OLD-1 and FLOR-1 are required in the epidermis for the induction of ORR genes. (*A*) Z stacks showing *old-1* and *flor-1* mRNA distribution by smFISH. *old-1* and *flor-1* mRNAs are shown as white spots, epidermal nuclei are marked by a *dpy-7p::GFP::H2B* transgene. (Scale bar is 10 µm.) Lower panel shows average fluorescent intensity of mRNA signal for *old-1* and *flor-1* along a normalised body length (n = 10 animals for each, error bars show SE for each bin). (*B*) An OLD-1::GFP *(icbEx310)* translational fusion is expressed primarily in the anterior epidermis, whereas a FLOR-1::GFP translational fusion *(icb136)* is expressed throughout the epidermis. Lower panels show that both translational fusions localize to the membrane in the head (white arrows). Scale bar for top four images is 50 µm, and for bottom zoomed-in images is 20 µm. (*C*) Epidermal-specific RNAi knock-down of *old-1* and *flor-1* expression is sufficient to reduce response to *M. humicola* extract to the same level as whole-body RNAi knock-down, n > 50 for each treatment. All treatments are significant with a chi-square test, ****P < 0.0001. (*D*) Overexpression of *old-1* under the *dpy-7* promoter (*icbEx309*) triggers constitutive activation of *chil-27p::GFP (*n > 100 animals, Scale bar is 100 µm). (*E*) Heatmap showing relative expression of the 50 most consistently up-regulated ORR genes upon epidermal overexpression of *old-1(icbEx359)* and extract-treated wild-type animals carrying hygromycin resistance (*icbEx360*). Significant (*q* value < 0.1, *P* value < 0.01) log2 fold changes are shown in blue, while white represents nonsignificance. (*F*) Representative survival assay for epidermal overexpression of *old-1*(*icbEx359*) which decreases susceptibility to infection by *M. humicola.* The control used for this experiment also carries the hygromycin resistance (*icbEx360*), n = 80 to 90 animals per condition, representative graph of three repats is shown, log rank test ***P < 0.001.

To determine in which tissue *old-1* and *flor-1* act, we performed single-molecule fluorescence in situ hybridization (smFISH). For both genes, we observed epidermal expression as evidenced by the proximity of detected mRNAs with epidermal GFP-labeled nuclei (*SI Appendix*, Fig. S2*A*). Interestingly, despite the *C. elegans* epidermis being a multinucleated syncytium (23), *old-1* showed higher expression in the anterior end of the epidermis, while *flor-1* mRNAs were more abundantly distributed throughout the epidermis (Fig. 2*A* and *SI Appendix*, Fig. S2*A*). Translational reporters created by the fusion of OLD-1 and FLOR-1 with GFP also matched the mRNA expression and revealed membrane localization in the epidermis (Fig. 2*B* and *SI Appendix*, Fig. S2*B*). Coexpression of OLD-1::GFP and FLOR-1::mScarlet showed membrane colocalization in the anterior epidermis (*SI Appendix*, Fig. S2*C*). As both OLD-1 and FLOR-1 colocalize in the epidermal membrane, we wished to determine whether they could be physically interacting to facilitate signaling. To this end, we used fluorescent resonance energy transfer (FRET) and found OLD-1::GFP and FLOR-1::mScarlet in close proximity to allow interaction both in the presence and absence of *M. humicola* extract (*SI Appendix*, Fig. S2*D*). Finally, we tested whether epidermis-specific RNAi targeting *old-1* or *flor-1* would be sufficient to prevent the induction of *chil-27p::GFP* upon extract treatment. We found that epidermal RNAi was sufficient to decrease *chil-27p::GFP* induction to the same extent as whole-body RNAi (Fig. 2*C*). Taken together, we conclude that OLD-1 and FLOR-1 are likely to act together in the epidermis.

Next, we tested whether we could mimic the constitutive activation of *chil-27p::GFP* seen upon overexpression of *old-1*, using the *dpy-7* promoter that drives expression specifically in the epidermis. Here, we found that *chil-27p::GFP* was expressed constitutively throughout the entire epidermis (Fig. 2*D* and *SI Appendix*, Fig. S3) as opposed to only the anterior side that is observed upon extract treatment. This difference in *chil-27p::GFP* expression is consistent with the observed *old-1* mRNA distribution either in the anterior epidermis of transgenics carrying extra copies of *old-1* under their endogenous promoter or throughout the epidermis when the *dpy-7* promoter was used instead (*SI Appendix*, Fig. S3). RNAseq analysis of animals overexpressing *dpy-7p::old-1* showed upregulation of >95% of ORR genes (Fig. 2*E* and *SI Appendix*, Dataset S2). Consequently, these animals also showed increased survival upon infection by *M. humicola* (Fig. 2*F*).

We have shown previously that activation of the ORR upon oomycete exposure is modulated by cross-tissue communication from neurons to epidermis, and TAX-2/4-dependent signaling in chemosensory neurons is able to modulate the induction of the ORR (12) as well as regulate cuticle morphology and epidermal pathogen avoidance (24, 25). Therefore, we asked whether activation of the ORR upon overexpression of *old-1* also requires neuronal signaling. Here, we found that constitutive expression of *chil-27p::GFP* upon overexpression of *old-1* is not suppressed by *tax-4* loss-of-function, further suggesting that OLD-1 acts at the level of the epidermis, thus downstream of neuronal signaling in the pathway leading to the induction of the ORR (*SI Appendix*, Fig. S4).

### FLOR-1 Is a Pseudokinase that Regulates OLD-1 Membrane Availability and Its Downstream Signaling.

**Fig. 3.**
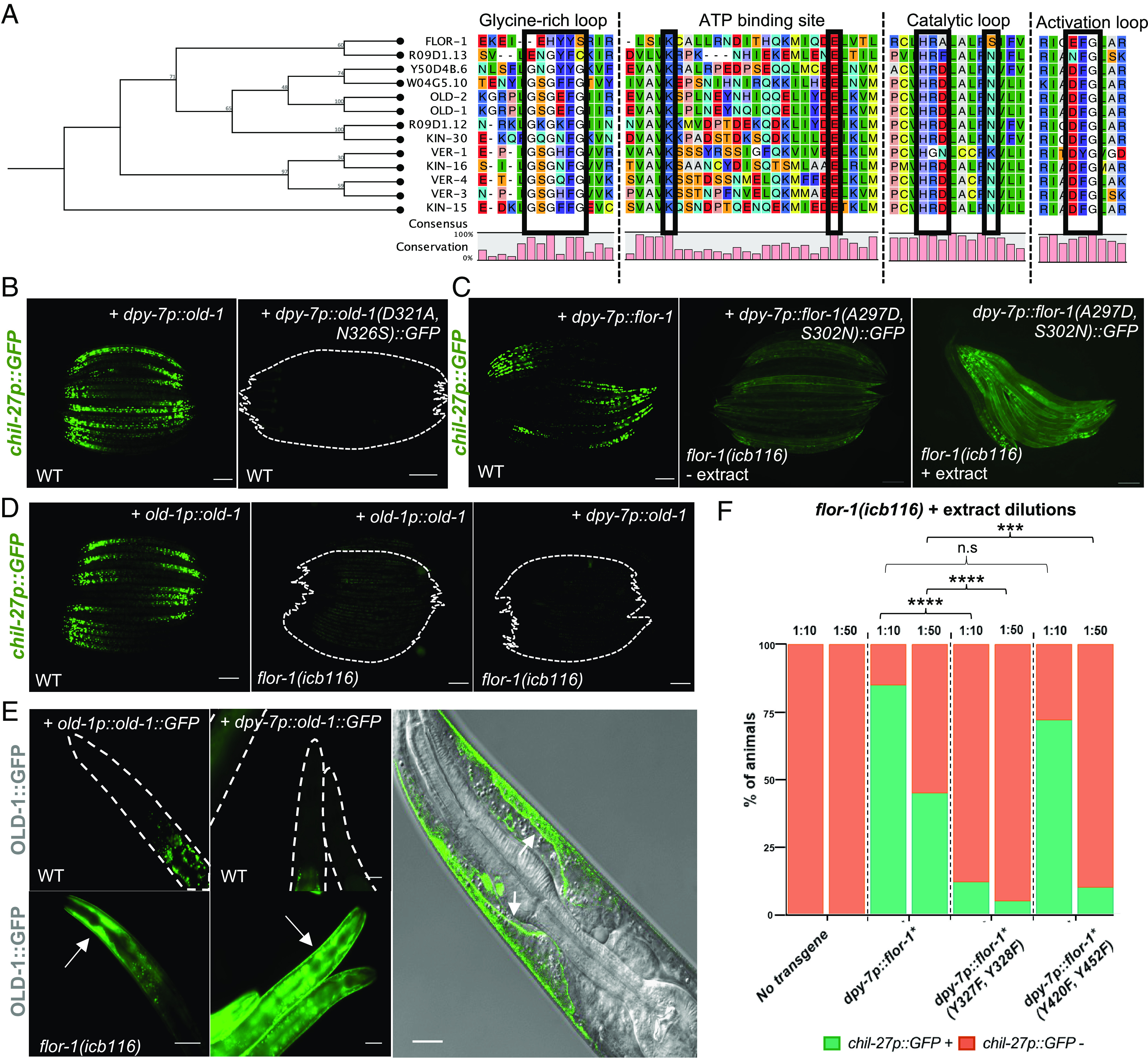
OLD-1 and FLOR-1 form a kinase–pseudokinase pair in the epidermal membrane. (*A*) Phylogenetic tree of the KIN-16 family of receptor tyrosine kinases focusing on alignment of key regions within the kinase domain to illustrate that FLOR-1 lacks conserved kinase domain features. Bootstrap values are shown at nodes of the tree. Protein sequences were aligned, and phylogenetic tree was created using CLC Sequence Viewer (Qiagen). (*B*) OLD-1(D321A, N326S) is unable to induce *chil-27p::GFP* upon epidermal overexpression *(icbEx316)*. (*C*) FLOR-1(A297D, S302N) is unable to induce *chil-27p::GFP* seen upon epidermal overexpression *(icbEx426)* but can rescue the response to extract in *flor-1(icb116)* mutants. The weak background GFP signal in the second panel represents FLOR-1 expression which is distinct from *chil-27p::GFP*. (*D*) Constitutive activation of *chil-27p::GFP* upon overexpression of *old-1p::old-1 (icbEx433)* and *dpy-7p::old-1 (icbEx434)* requires FLOR-1. For *B*–*D*, n > 100 and Scale bar is 100 µm. (*E*) Expression of *OLD-1::GFP (icbEx414, icbEx436*) is increased in the absence of *flor-1* (n > 100 animals, Scale bar is 50 µm). Note that *old-1* is expressed at low levels so the OLD-1::GFP signal is not visible in wild type under these imaging conditions (GFP signal represents autofluorescence from the intestine). Confocal image on the right shows that OLD-1::GFP localizes normally at the membrane of the epidermis in the head (white arrows) in *flor-1(icb116)* mutants. (Scale bar is 10 µm.) (*F*) Response to extract dilutions (1:10 & 1:50) quantified in *flor-1(icb116)* mutants overexpressing different mutant forms of phosphorylation sites in *flor-1* in the hypodermis (n = 50 per strain, ****P < 0.0001, ***P < 0.001 with chi-square test). Phosphorylation mutants were created in *flor-1* sequence which was already carrying A297D and S320N mutations (flor-1*).

Despite the fact that OLD-1 and FLOR-1 belong to the same RTK family, we saw consistently higher induction of *chil-27* upon overexpression of *old-1* rather than *flor-1* (Fig. 1*D* and *SI Appendix*, Fig. S1). Upon closer investigation, we noticed amino acid changes in key regions in the FLOR-1 protein sequence, such as the glycine-rich, activation and catalytic loop (26, 27), which are likely to interfere with its kinase activity (Fig. 3*A*). For example, a characteristic signature of catalytically deficient kinases, also known as pseudokinases, is mutations in an aspartate (generally referred to as “D166”) and asparagine (“N171”), present within a HxDxxxxN motif required for catalysis. This motif was present in OLD-1 (HRDLALRN) and other family members, but not in FLOR-1 (HRALALRS). We used site-directed mutagenesis to mutate the D and N residues in the catalytic loop of OLD-1 to those amino acids found in FLOR-1 (A and S respectively). This conversion resulted in loss of constitutive activation of *chil-27p::GFP*, suggesting that an active kinase function is required for ligand independent signaling upon overexpression of *old-1* (Fig. 3*B* and *SI Appendix*, Fig. S5). Unlike FLOR-1 overexpression under its endogenous promoter that does not significantly induce *chil-27* expression, animals expressing high levels of FLOR-1 via the *dpy-7::flor-1* transgene show constitutive expression of *chil-27* even in the absence of extract treatment (Fig. 3*C* and *SI Appendix*, Fig. S5). However, when the same OLD-1-like catalytic residues were introduced in FLOR-1 (A297D, S302N), the induction of *chil-27p::GFP* was reduced, while FLOR-1 retained the ability to rescue *chil-27p::GFP* induction in *flor-1(icb116)* mutants upon exposure to oomycete extract (Fig. 3*C* and *SI Appendix*, Fig. S5). These results indicate that FLOR-1 is likely to be a pseudokinase with reduced or no active kinase activity.

Next, we sought to determine the relationship between the two RTKs. To this end, we expressed *old-1* both under its endogenous promoter and under the epidermal-specific *dpy-7* promoter in a *flor-1(icb116)* mutant background. In both cases, the constitutive expression of *chil-27p::GFP* was no longer observed (Fig. 3*D*), suggesting that FLOR-1 is required for OLD-1-mediated signaling. To determine the expression and localization of OLD-1 in the absence of *flor-1*, we expressed OLD-1::GFP in a *flor-1(icb116)* mutant. The expression of OLD-1 was generally low, even upon transgene overexpression at high concentrations as part of multicopy arrays using strong epidermal promoters (*dpy-7p*) (*SI Appendix*, Fig. S6*A*). Low OLD-1 levels are likely to reflect proteasomal regulation because treatment with the proteasome inhibitor bortezomib (BTZ) led to an increase in OLD-1 abundance (*SI Appendix*, Fig. S6 *A* and *B*). Surprisingly, in *flor-1(icb116)* mutants, we saw high levels of GFP expression with correct membrane localization in the anterior epidermis when OLD-1 was expressed under its endogenous promoter (*old-1p::old-1::GFP*), and expression throughout the epidermis when OLD-1 was expressed under an epidermis-specific promoter (*dpy-7p::old-1::GFP)* (Fig. 3*E*). Furthermore, an independent ethyl methansulfonate (EMS) suppressor screen performed on animals constitutively expressing OLD-1::GFP *(icbIs22)* identified five further alleles of *flor-1* fully suppressing the constitutive activation of *chil-27p::GFP* while causing accumulation of OLD-1::GFP in the anterior epidermis (*SI Appendix*, Fig. S6*C*). These results substantiate the requirement of FLOR-1 for regulating the downstream signaling pathway and OLD-1 levels at the epidermal membrane.

Given that FLOR-1 appeared to act downstream of OLD-1, we next asked whether OLD-1 could activate the downstream signaling pathway by phosphorylating FLOR-1. To answer this, we mutated specific tyrosine residues in FLOR-1 to phenylalanine and tested their ability to rescue *chil-27p::GFP* induction in *flor-1(icb116)* mutants upon oomycete extract treatment. We chose to mutate the two adjacent tyrosines near the activation loop (Y327 and Y328) and two tyrosines (Y420 and Y452) toward the C-terminal end of FLOR-1. The rationale here was that activation loop tyrosine residues have been previously shown to be important for the function of pseudokinases (28), and a *flor-1(icb126)* allele recovered from our OLD-1::GFP suppressor screen was predicted to produce a truncated protein lacking tyrosines Y420 and Y452. We used FLOR-1(A297D, S302N) as the template for mutagenesis to avoid inducing *chil-27p::GFP* constitutively. While FLOR-1 overexpression was strongly able to rescue *chil-27p::GFP* induction in *flor-1(icb116)* upon extract treatment, the response obtained with FLOR-1(Y327F, Y328F) and to a lesser extent with FLOR-1 (Y420F, Y452F) was significantly reduced (Fig. 3*G*). These results suggest that OLD-1-mediated phosphorylation of multiple FLOR-1 tyrosine residues is likely to be required for the activation of downstream signaling pathway, with the phosphorylation of tyrosine residues near the activation loop being more critical than the others.

### The Transcription Factor VAB-3 Regulates *old-1* to Activate the ORR.

**Fig. 4.**
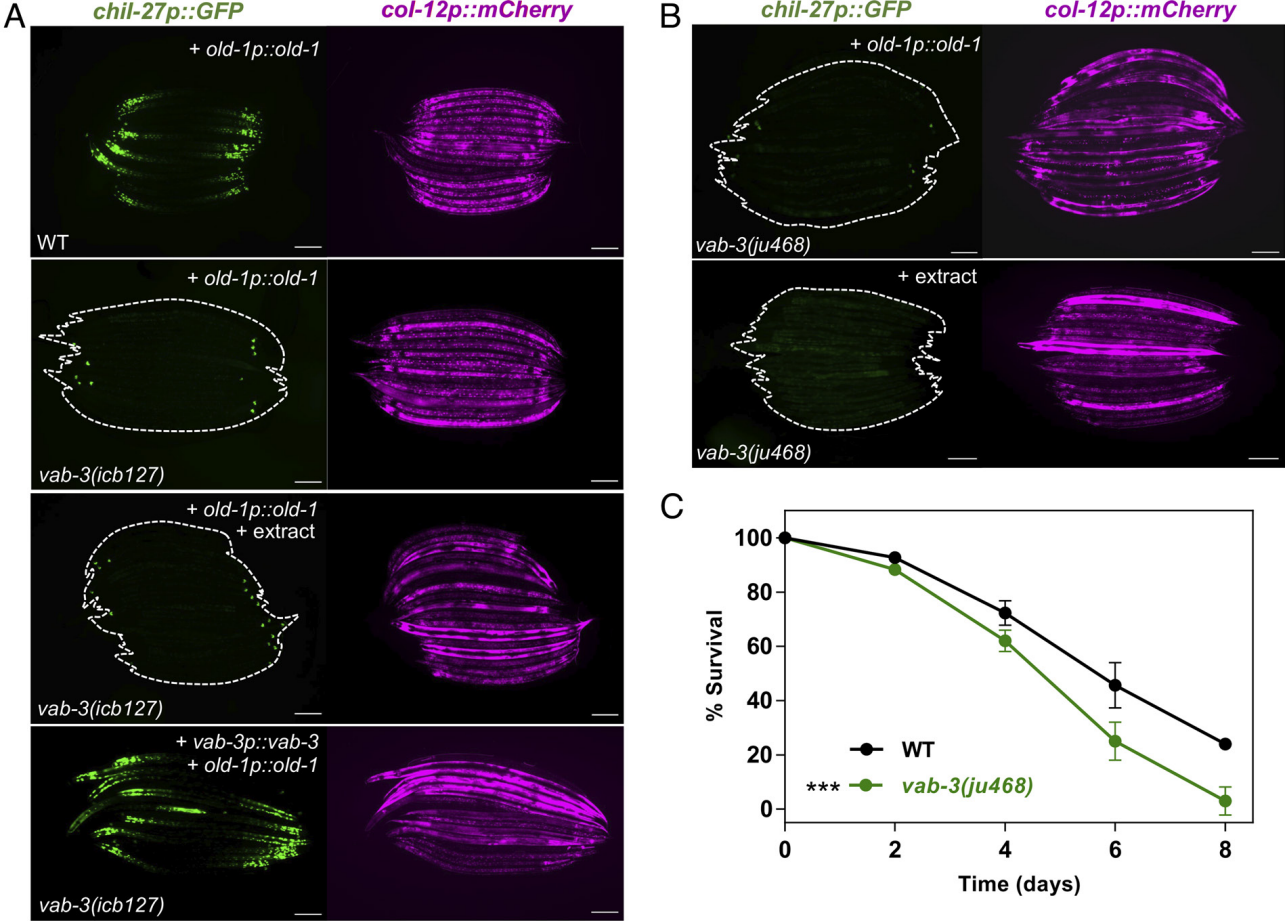
PAX-6 homolog VAB-3 regulates activation of ORR. (*A*) A mutation in *vab-3(icb127)* inhibits constitutive activation of *chil-27p::GFP* and induction of *chil-27p::GFP* in response to *M. humicola* extract. Constitutive expression of *chil-27p::GFP* was restored in *vab-3(icb127)* carrying an *old-1p::old-1* transgene by overexpressing a *vab-3* containing fosmid (WRM0640cC05). (*B*) *vab-3(ju468)* mutant phenocopies loss of *chil-27p::GFP* induction upon extract treatment as well as upon *old-1* overexpression. (*C*) *vab-3(ju468)* mutants are hyper-susceptible to infection by *M. humicola* (n = 90 per condition, representative graph of three repeats shown) ***P < 0.001 Log-Rank test.

One of the recovered mutations suppressing the *old-1* overexpression phenotype involved a substitution (R269Q in *icb127*) within the homeodomain (HD) of the transcription factor VAB-3. VAB-3 is the *C. elegans* PAX6 homolog and is well studied for its role in head morphogenesis and cell fate specification in anterior epidermal cells (29, 30). The *vab-3(icb127)* mutation caused loss of constitutive *chil-27p::GFP* expression upon *old-1* overexpression as well as loss of *chil-27p::GFP* induction upon exposure to the oomycete extract (Fig. 4*A*). Rescue with a wild-type copy of *vab-3* restored constitutive *chil-27p::GFP* expression upon *old-1* overexpression in *vab-3(icb127)* (Fig. 4*A*). To confirm that *icb127* is a new allele of *vab-3*, we also tested *vab-3(ju468)* strong loss-of-function mutants carrying a nonsense mutation (Q262STOP) within the HD of the protein (Fig. 4*A*). We found that the *vab-3(ju468)* mutation suppressed *chil-27p::GFP* induction both upon overexpression of *old-1* and extract treatment (Fig. 4*B*). Consequently, *vab-3(ju468)* mutants were found to be hypersusceptible to *M. humicola* (Fig. 4*C*). These results suggest that *vab-3* is a key regulator of the response to oomycete recognition.

Besides the HD, PAX6 isoforms can include an additional DNA binding domain, known as the paired domain (PD), and there is evidence that isoforms with or without the PD can have separable expression and functions (31). As *icb127* and *ju468* mutations map within the HD, they affect all isoforms of the protein. To test whether *chil-27p::GFP* induction is influenced by VAB-3 specifically through the HD, we tested another previously described *vab-3* allele (*wz25*) that involves a substitution (G52R) in the PD leaving a functional HD. We found that *vab-3(wz25)* mutants show normal induction of *chil-27p::GFP* upon exposure to oomycete extract (*SI Appendix*, Fig. S7). This suggests that activation of the ORR is dependent on the HD of VAB-3.

**Fig. 5.**
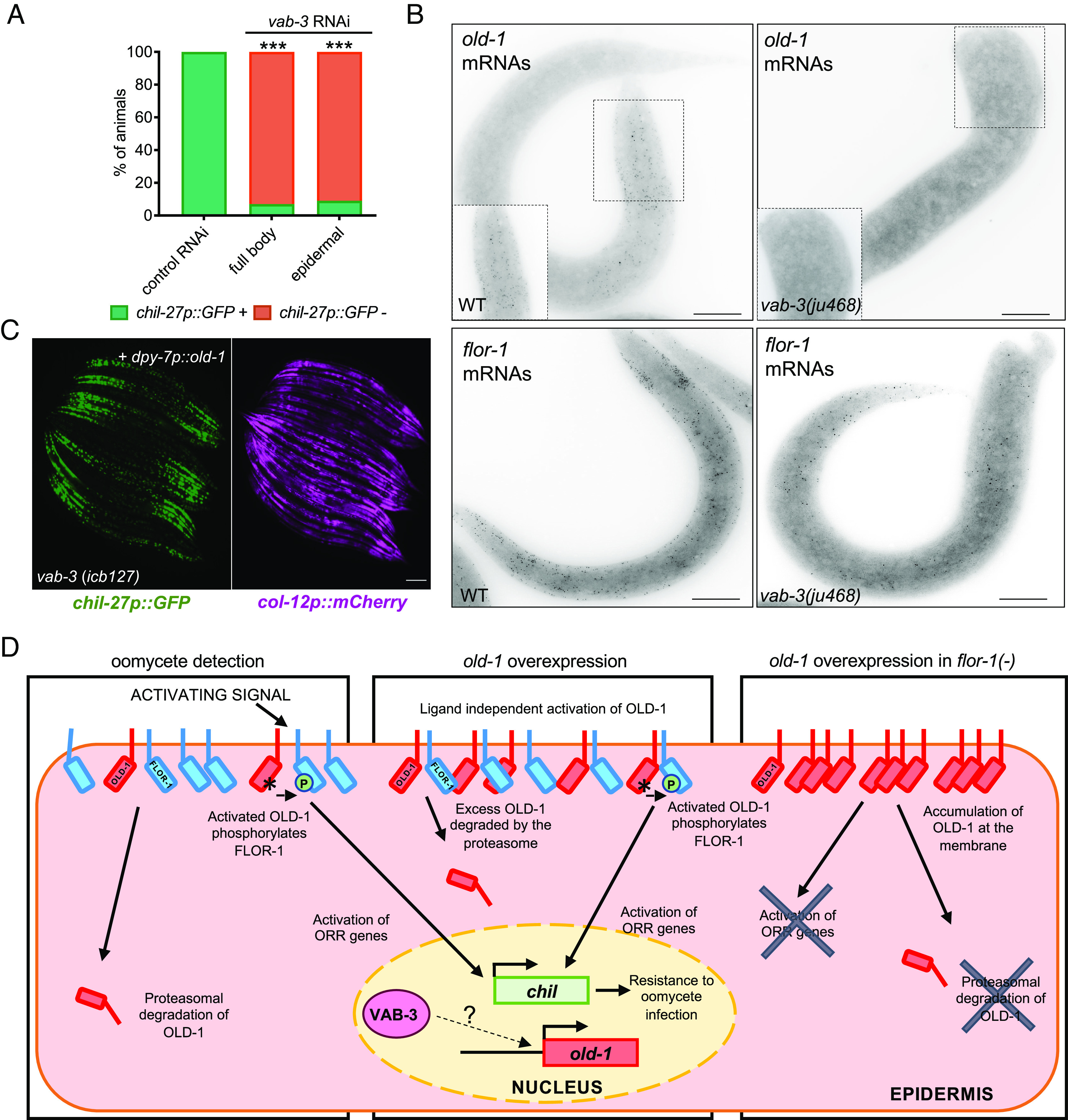
VAB-3 regulates *old-1* expression. (*A*) Epidermal-specific RNAi knock-down of *vab-3* expression is sufficient to reduce response to *M. humicola* extract to the same level as whole-body RNAi knock-down, n > 50 animals for each treatment. Stars represent significance with a chi-square test, ***P < 0.001. (*B*) smFISH for *old-1* and *flor-1* expression in WT animals and *vab-3* mutants. Scale bar is 10 µm, n = 15. (*C*) Constitutive activation of *chil-27p::GFP* is observed in *vab-3(icb127)* upon overexpression of *dpy-7p::old-1*, n > 100 animals and Scale bar is 100 µm. (*D*) Schematic model of the proposed interaction between OLD-1 and FLOR-1 upon oomycete detection, *old-1* overexpression and in a *flor-1(-)* mutant background. FLOR-1 is both required to facilitate signaling of the oomycete recognition response possibly through phosphorylation by OLD-1 and for regulating availability of OLD-1 at the membrane. VAB-3 modulates *old-1* expression likely in an indirect manner (shown with question mark) and thus influences the activation of ORR.

To investigate the expression pattern of *vab-3,* we used smFISH and found localization of *vab-3* mRNAs in the anterior epidermis, as expected based on previous reports (29) (*SI Appendix*, Fig. S8). Notably, the *vab-3* expression pattern is reminiscent of *old-1* expression (*SI Appendix*, Fig. S8); therefore, we asked whether VAB-3 function is required in the epidermis for activation of the ORR and whether the role of VAB-3 might involve regulation of *old-1* or *flor-1* expression. We performed epidermis-specific RNAi of *vab-3* and found significant reduction in expression of *chil-27p::GFP* upon extract treatment (Fig. 5*A*). Using smFISH and qRT-PCR, we found a significant reduction of *old-1* expression in *vab-3(ju468)*, while *vab-3(wz25)* animals showed comparable *old-1* levels to wild type (Fig. 5*B* and *SI Appendix*, Fig. S9). Instead, *flor-1* levels remained unchanged in all *vab-3* mutants (Fig. 5*B* and *SI Appendix*, Fig. S9). Furthermore, we overexpressed *old-1* in *vab-3(icb127)* under an epidermis-specific *dpy-7* promoter to circumvent the potential requirement for VAB-3 regulation and found that the *vab-3* mutation no longer suppressed the constitutive *chil-27p::GFP* expression (Fig. 5*C*). Taken together, these results suggest that VAB-3 regulates *chil-27* induction through *old-1* expression.

Finally, we sought to determine whether VAB-3 is able to directly regulate the transcription of *old-1*. We noticed that within the promoter sequence of *old-1*, there are predicted HD binding sites (TAAT(N_5_)ATTA) at −472 nucleotides upstream of the TSS (*SI Appendix*, Fig. S10). Such palindromic DNA sequences can be bound by the HD of PAX6 in vitro (32). We used CRISPR to delete the TAAT(N_5_)ATTA sequence in the *old-1* promoter but found no consequence on *old-1* expression and *chil-27p::GFP* induction upon exposure to extract (*SI Appendix*, Fig. S10). This suggests that VAB-3 is likely to regulate expression of *old-1* through another intermediate factor or binding to noncanonical regulatory elements.

To conclude, these results allow us to propose a model where OLD-1 is an epidermal RTK whose expression is influenced by VAB-3 and membrane localization regulated by another RTK family member FLOR-1. When OLD-1 is activated upon oomycete exposure or overexpression, it can likely phosphorylate FLOR-1 and trigger the activation of ORR and thus oomycete resistance (Fig. 5*D*).

## Discussion

Building upon our previous discovery of cross-tissue signaling in the oomycete recognition response in *C. elegans* (12), we present here two key regulators of this program, namely OLD-1 and FLOR-1. These are members of the KIN-16 family of receptor tyrosine kinases (18) and are necessary to drive the epidermal component of the defence response against oomycete infection. While *old-1* has been implicated in lifespan extension and stress resistance (33), there has been no previous link to immunity. We report that several members of this family, such as *kin-15*, *old-2, ver-1,* and *R09D1.12* are induced as part of the ORR, however, both *old-1* and *flor-1* expression is not induced upon infection, so these new regulators could not have been identified through transcriptomic profiling. This is similar to what has been described for other gene families and immune programmes like the *pals* genes and IPR, where some *pals* genes are part of the IPR, while other *pals* family members regulate the induction of IPR (34). It is also interesting that the *old-1* locus as well as several other members of the KIN-16 family, such as *kin-15, kin-16, R09D1.12, old-2,* and *R09D1.13,* are located within the previously identified *chil* gene clusters on chromosome II (8). Genomic clustering of *C. elegans* immune response genes has been previously reported in other contexts, for example, with regard to genes induced in response to *Microbacterium nematophilum* (35), the *nlp* and *cnc* gene clusters up-regulated in response to the fungus *Drechmeria coniospora* (36), or the *pals* gene clusters containing multiple *pals* members (34). As *old-1* regulates *chil* gene expression to antagonize infection by *M. humicola,* our study highlights that the close proximity between KIN-16 family members and *chil* genes might be indicative of a cluster of resistance genes acting in the same signaling pathway underlying oomycete-specific immunity.

Alignment of all members of the KIN-16 family revealed that FLOR-1 contains key amino acid changes in highly conserved regions that are expected to be required for full kinase activity. Further, 10% of human RTKs are thought to be missing at least one conserved, catalytically important residue and are thus classified as putative pseudokinases (26). First, the glycine loop with the consensus sequence GxGxxG, which plays a role in phosphate binding, is missing in FLOR-1. Substitution of the first two glycines is thought to distort the ATP binding site (27). Second, the catalytic loop of RTKs contains two conserved residues—the aspartate in the HRD motif is thought to function as the catalytic base for phosphorylation and an asparagine is required for metal ion binding for catalysis (27). FLOR-1 does not have either of these conserved amino acids. Third, the VAIK motif coordinates the co-factor ATP, anchoring and orienting the ATP and forming a salt bridge with E91 (37). Although the K/E residues are present in FLOR-1, the motif differs from the consensus in the family (LSIK vs. VAVK) and may hinder ATP binding. Finally, the DFG (Aspartate, Phenylalanine, Glycine) motif of the activation loop has also diverged from the consensus in the family—the aspartate is thought to be one of the most critical amino acids for active kinase function as it is required for divalent cation binding required for catalysis (27). FLOR-1 does not have the conserved aspartate in this motif, instead, it has glutamate, a substitution that is sufficient to inactivate phospho-transfer in kinases (38–40). In summary, while a single change in any of these conserved motifs is usually sufficient for putative classification as a pseudokinase, FLOR-1 has changes in at least four out of these six critical amino acids. Nevertheless, FLOR-1 is functional and biologically important because it is required for immune signaling upon oomycete recognition acting downstream of the OLD-1 receptor tyrosine kinase.

The identification of FLOR-1 playing a role in oomycete resistance expands the repertoire of known pseudokinases associated with immune signaling in *C. elegans*. For example, the putative pseudokinase *nipi-3* acts as a negative regulator of the transcription factor *cebp-1* and a positive regulator of *skn-1* in the intestinal immune response against *Pseudomonas aeruginosa* (41, 42). *nipi-3* is also necessary for the *D. coniospora*-specific upregulation of *nlp-29* that triggers the p38-signaling cascade (6). *nipi-3* is part of the Tribbles pseudokinase family and is thought to have lost the active kinase function, acting instead as a scaffold or adaptor protein involved in the assembly or regulation of signaling components (43). Part of the same family, *nipi-4* is a pseudokinase also involved in the positive regulation of *nlp* genes upon *D. coniospora* infection, in addition to controlling the constitutive expression of antimicrobial *cnc* peptides that are regulated by the TGF-β pathway (44).

It is thought that some pseudokinases, despite their unusual sequence features, may still be able to catalyze phosphotransfer albeit to a more limited extent (27, 37, 45). For example, HER3 (human epidermal growth factor receptor 3, also known as ErbB3) is an example of a low-activity kinase. It is one of four members of the human epidermal growth factor receptor tyrosine kinase family but stands out as the only member to have changes in the conserved ATP binding region (46). This family is well studied due to its implication in tumorigenesis and is also a target for several anticancer drugs (47). HER3 lacks the glutamate in the ATP binding region (substituted for a histidine) and replaces the aspartate in the HRD motif of the catalytic loop with an asparagine, leading to the initial hypothesis that HER3 may be catalytically inactive (48). However, follow-up studies have shown that HER3 is in fact able to bind ATP and is capable of trans-autophosphorylation (49, 50). Interestingly, this activity is much lower than other HER proteins and is dependent upon its active kinase partner, HER2 (49, 51). The weak kinase activity within a HER2/HER3 heterodimer regulates the active kinase partner, which then uses its increased kinase capabilities to phosphorylate substrates (49). While we cannot rule out that FLOR-1 may have some weak kinase activity, it appears that it is phosphorylated downstream of OLD-1. Therefore, FLOR-1 is more likely to serve as a scaffold for the assembly of signaling proteins leading to activation of the ORR. Phosphoproteomic-based identification of OLD-1 targets, together with further genetic and RNAi screens, can help elucidate in the future the tissue-specific signaling network involved downstream of OLD-1/FLOR-1 in response to oomycete recognition in *C. elegans*.

Our study also provides evidence for a link between the homeobox transcription factor VAB-3/PAX6 and the regulation of immune signaling in *C. elegans*. PAX6 is a highly conserved transcription factor, critical for its role in visual system development, and mutations in PAX6 are known to be associated with a wide array of symptoms, most notably congenital eye malformations (52, 53). Other Hox genes have been previously shown to modulate immune responses in *C. elegans* (54, 55). An intestinal homeobox gene in *Drosophila* has been shown to repress nuclear factor kappa B-dependent antimicrobial peptide expression (56). Our results suggest that a conserved developmental regulator of anterior epidermal morphogenesis also regulates the expression of a key component required for the oomycete recognition response, thereby shaping the spatial pattern of immune signaling activation. It is of note that the *vab-3* and *old-1* expression gradient from the anterior epidermis to the posterior body can readily explain the characteristic gradient of *chil-27* induction upon pathogen recognition. While our results suggest that the VAB-3-mediated regulation of *old-1* expression may not be direct, it is more likely to rely on intermediate factors rather than be a consequence of broader changes in epidermal cell identity because other epidermal genes, such as *flor-1* and *col-12,* are expressed normally in *vab-3* mutants lacking *old-1* expression. It would be interesting to explore in the future whether PAX6 is required for shaping immune responses to infection by oomycetes or other natural pathogens in different animal contexts.

## Methods

### *C. elegans* and *M. humicola* Maintenance.

All *C. elegans* strains (listed in *SI Appendix*, Table S1) were cultured and maintained on NGM plates seeded with *Escherichia coli* OP50 under standard conditions (57). *M. humicola* was maintained by chunking infected worms onto fresh NGM plates and supplementing with fresh N2. For use in experiments, dead animals filled with sporangia were picked and transferred to the *E. coli* lawn of fresh NGM plates.

### EMS Mutagenesis.

Animals carrying the *chil-27p::GFP* reporter (*icbIs5)* were mutagenized using the chemical mutagen ethyl methanesulfonate (Sigma). L4 stage animals (P0) were incubated with 24 mM EMS in 4 mL M9, for 4 h. Worm pellets were washed 10 times with 15 mL M9 to remove residual EMS. F2s were treated with extract and animals no longer expressing the *chil-27p::GFP* reporter were selected. Similarly, for the *old-1p::old-1* suppressor screen, F2 animals no longer constitutively expressing *chil-27p::GFP* were selected. Around 60,000 and 20,000 haploid genomes were screened, respectively, to identify suppressor mutants. *old-1* and *flor-1* mutants were mapped by crossing to the polymorphic CB4856 strain, and progeny from 15 to 25 F2 recombinants was pooled in equal proportion to obtain genomic DNA for whole-genome sequencing of each independent mutant. Sequencing was performed by Novogene (Cambridge) and WGS data were analyzed using the CloudMap Hawaiian variant mapping pipeline to identify causative mutations (58, 59) and MIModD (https://mimodd.readthedocs.io/en/latest/index.html). In brief, variant allele frequency was mapped by first aligning the mutant genome sequence to N2 reference genome, followed by a comparison with single-nucleotide polymorphisms found in CB4856. Regions enriched for N2 were then analyzed for candidates, which were homozygous mutations not found in the CB4856 background.

### Infection and Induction Assays.

Infection assays were performed in triplicate at 20 °C on NGM plates seeded with 100 µL OP50. Five *M. humicola-*infected (dead) animals were added to the OP50 lawn of four plates and minimum of 20 L4s were transferred to each plate, meaning a minimum of 80 animals were used per biological repeat. Dead animals with visible sporangia were scored every 48 h, and live animals were transferred to a new NGM plate supplemented with an additional five *M. humicola-*infected (dead) animals. Dead animals without evidence of infection or missing animals were censored from the counts. The figures show representative graphs of three biological replicates with a minimum of 80 animals per biological replicate.

*M. humicola* extract was made and induction assays were performed as described previously (12). *M. humicola* extract was added to the OP50 lawn when *C. elegans* eggs were added to the plate or at L2 stage. The presence or absence of *chil-27p::GFP* was quantified at L4 or day-1 adult stage using a Zeiss Axio Zoom V16 microscope.

### RNA-Sequencing.

For all RNA-seq experiments, animals were synchronized via bleaching and collected in triplicate for RNA extraction at L4 stage. Where extract was used, 200 µL was added to the OP50 lawn 4 h before animal collection. Total RNA was extracted using TRIzol (Invitrogen) and transcriptome sequencing was completed by BGI (Hongkong) and Novogene (Cambridge). Kallisto (60) was used for alignments with the WS283 transcriptome from Wormbase. Count analysis was performed using Sleuth (61) along with a Wald Test to calculate log_2_fold changes. All RNAseq data files are publicly available from the NCBI Gene Expression Omnibus (GEO) database under the accession number GSE220958.

### Microscopy.

Single-molecule FISH and image analysis was performed as described previously (62). In brief, embryos collected by treating gravid adults with a standard solution containing sodium hypochlorite were grown at 20 °C until L2 stage when they were fixed using 4% formaldehyde (Sigma-Aldrich) for 45 min and permeabilized with ethanol for at least 24 h. Samples were then hybridized with a probe for 16 h (probe sequences included in *SI Appendix*, Table S2). Imaging was performed using the 100× objective of a Nikon Ti-eclipse inverted microscope fitted with an iKon M DU-934 CCD camera (Andor) and operated via the Nikon NIS Elements Software. Spot quantification was performed using MATLAB. For quantification of smFISH signal using FIJI, Z-slices with visible mRNAs were max-projected to give a single image. This image was then adjusted using an auto-threshold to binarize the image and a segmented line was drawn along the length of the animal. To calculate the fluorescent intensity along the length of the segmented line, the “plot profile” plugin was used. This analyzed fluorescent intensity per μm of animal length was then normalized into 100 bins. The curve was smoothed using Prism (GraphPad) to see the trend of expression across the length of the epidermis.

Confocal microscopy was performed on a Leica SP8 inverted confocal microscope with animals anesthetized using 10 mM sodium azide and mounted on a 2% agarose pad. Prior to FRET imaging, mixed-stage animals were fixed using 4% formaldehyde (Sigma-Aldrich) for 45 min and mounted on a 2% agarose pad. Where extract was used, 200 µL was added to the plate 24 h before fixation. Microscopy was performed using a Leica Stellaris 5 in Confocal mode to perform acceptor depletion FRET at 63× magnification. GFP and mScarlet expression were measured three times prior to bleaching using a 488-nm and 561.4-nm laser, respectively. mScarlet was then photobleached using the 561.4-nm laser, and GFP and mScarlet expression were measured three times post photobleaching. Change in fluorescent intensity was analyzed using FIJI macros developed by the Imperial College FILM facility.

### Molecular Cloning and Transgenesis.

For rescue of mutant phenotypes and initial overexpression of *old-1*, gene sequences were PCR amplified from N2 genomic DNA using primers old-1FullF and old-1FullR and injected at 20 ng/µL. *old-1::GFP* was PCR amplified from the fosmid CBGtg9050E024D (TransgeneOME) using primers old-1FullF and old-1FullR and injected at 50 ng/µL. For rescue of *flor-1(icb116)*, the full gene locus was amplified from genomic DNA using primers T01G5.1promF and T01G5.1FullR2 and injected at 20 ng/µL. *flor-1::flor-1::GFP* was PCR amplified from MBA998 using the primers T01G5.1promF and T01G5.1FullR2 and injected at 30 ng/µL. The *flor-1::flor-1::mScarlet* plasmid (pER6) was created using Gibson cloning into a pER4 *(wrmScarlet::unc-54 3′UTR)* backbone using primers T01G5.1promF and T01G5.1R_pER2/4 to amplify the gene locus and injected at 40 ng/µL.

All constructs expressed under the *dpy-7* promoter were inserted into plasmid pIR6 digested with FastDigest enzyme SmiI, using Gibson Cloning. The *dpy-7p::old-1* plasmid (pFD20) was created by amplifying *old-1* from gDNA using primers dpy-7old-1gibsonfwd and dpy-7old-1gibsonrev. The *dpy-7p::old-1(D321A, N326S)::GFP* plasmid (pFD23) was created using a Site-Directed Mutagenesis Kit (Agilent) to introduce mutations into pFD21 using primers old-1kinasemutF and old-1kinasemutR. The *dpy-7p::flor-1::GFP* plasmid (pJS1) and the *dpy-7p::flor-1* plasmid (pJS2) were made using primers dpy-7T01G5.1gibF and T01G5.1unc-54 gibR to amplify *flor-1::GFP* and *flor-1* from MBA998 and gDNA, respectively. Site-directed mutagenesis was carried out on a *dpy-7p::flor-1::GFP* plasmid (pJS1) to introduce key OLD-1 catalytic residues back into FLOR-1. The resulting *dpy-7p::flor-1(A297D, S302N)::GFP* plasmid (pJS4) was made using primers T01G5.1 SDM F and T01G5.1 SDM R. To mutate specific tyrosines in FLOR-1, site-directed mutagenesis was carried out on the plasmid pJS4. Residues Y327 and Y328 in the FLOR-1 activation loop were mutated using primers FLOR-1 SDM AL F2 and FLOR-1 SDM AL R2 to generate the dpy-7p::flor-1*(Y327F, Y328F)::GFP plasmid (pJS6). Residue Y420 in FLOR-1 was mutated using primers T01G5.1 Y420 SDM F and T01G5.1 Y420 SDM R. Residue Y452 in FLOR-1 was subsequently mutated using primers T01G5.1 Y452 SDM F and T01G5.1 Y452 SDM R to generate the dpy-7p::flor-1*(Y420F, Y452F)::GFP plasmid (pJS7). All constructs under the *dpy-7* promoter were injected at 5 ng/µL. Mutants carrying the *icb127* allele were rescued using injection with fosmid WRM0640cC05 (Source BioScience) at 10 ng/µL.

The coinjection markers used for transgenesis included a plasmid for hygromycin selection pDNRHyg (*rps-0p::hygB:unc-54*) (8), pRJM163 (*bus-1p::GFP*) (gift from the McMullan lab), and pWD47 (*myo-2p::DsRed)* (63) along with pBJ36 (64) as carrier DNA. The genotypes of all strains generated and sequences of all the oligos used can be found in *SI Appendix*, Table S2.

### CRISPR-Mediated Genome Editing.

The endogenous copy of *flor-1* was tagged with codon optimized GFP (GFPo) at the C-terminus using a CRISPR/Cas-9 GFP approach. GFPo was amplified from plasmid pDK57 using two sets of primers: GFPoATG-F and GFPoSTOP-R and T01G5.1Frep and T01G5.1Rrep, which carry 120 bp homology either side of the Cas-9 cut site. Equimolar quantities of these two PCR products were melted and reannealed to create a repair template with a single-stranded homology on each arm. The repair template was injected at 0.11 ng/µL into wild-type animals along with 0.25 ng/µL protein Cas-9 (IDT), 0.02 ng/µL tracrRNA (IDT), 0.02 ng/µL crRNA (IDT), and 40 ng/µL *rol-6 (su1006)* (65). Roller animals were selected and genotyped using primers T01G5.1fwd and GFPoSTOPR. The repair template introduced three synonymous changes at the end of the *flor-1* coding sequence to prevent cutting by Cas9. Similarly, to delete the Hox binding site in *old-1* promoter, N2 animals were injected with 4.76 µM crRNA (target sequence TTTCATTAATTGACAATTAG) (RMDW0838, IDT), 0.5 µM tracrRNA (IDT), 5.5 µM ultramer *old-1* repair template along with 5 ng/µL of *myo-2p::DsRed*, and 0.25 ng/µL protein Cas-9 (IDT). Transgenic animals with red pharynx were picked and genotyped using primers old-1_CRISP_F and old-1_CRISP_R. Deletion of Hox binding site was confirmed by sequencing with the primer old-1 full F.

### RNAi.

RNAi experiments were performed using the feeding method. The RNAi clones for *old-1* and *vab-3* were obtained from the Ahringer Library (Source BioScience) and were confirmed by sequencing prior to use. The RNAi clone for *flor-1* was custom made using Gibson cloning. A 1,179 bp *flor-1* fragment was PCR amplified from WT genomic DNA using primers TT01G5.1RNAiF and TT01G5.1RNAiR. The fragment was then cloned using Gibson assembly into pL4440 (Addgene) plasmid and transformed into *E. coli* HT115. Expression of dsRNA was induced by the addition on 1 mM IPTG (Sigma) to NGM plates. Either 6 L4 stage animals were added to RNAi plates and their progeny at L4 stage was scored after 72 h at 20 °C or embryos were added directly to RNAi plates and scored for *chil-27p::GFP* induction 48 h later. Where extract was used, 200 µL was added 48 h before scoring. For epidermis-specific RNAi, *chil-27p::GFP* reporter was crossed into the previously described strain QK52 (66).

### BTZ Treatment.

L2 stage animals were treated with 20 µM of Bortezomib (Sigma-Aldrich) or dimethyl sulfoxide (DMSO) (vehicle control) for 24 h followed by imaging at 20× magnification on a compound microscope (Zeiss). The GFP signal in the head region of 20 randomly picked animals was quantified using FIJI and corrected total cell fluorescence (CTCF) was calculated. Student’s *t* test was used to assess statistical significance.

### qRT-PCR.

Mixed-staged animals with 4-h extract treatment or without were collected in TRIzol (Invitrogen) and RNA was extracted using isopropanol/ethanol precipitation. The quantity and quality of RNA were estimated by NanoDrop quantification (Thermo Scientific) and gel electrophoresis, respectively. cDNA was synthesized using Superscript IV (Invitrogen) with Oligo(dT) primers using 2 to 3 µg RNA as per the manufacturer’s instructions. Real-time PCR was performed using qPCR primer pairs listed in *SI Appendix*, Table S2 and LightCycler480 SYBR Green I Master (Roche) in a LightCycler480 instrument. Ct values were derived using the LightCycler480 software and second derivative maximum method. All experiments were performed in biological triplicates. The expression levels of *old-1*, *flor-1*, and *chil-27* were normalized with the values obtained for the reference gene *pmp-3.* The efficiency of each set of primers and calculation of levels of induction was calculated by the 2^−ΔΔCt^ method.

## Supplementary Material

Appendix 01 (PDF)

Dataset S01 (XLSX)

Dataset S02 (XLSX)

## Data Availability

All study data are included in the article and/or *SI Appendix*. RNAseq files are publicly available from NCBI GEO database under accession number GSE220958 (67).
