# Supplementary material for: A PAX6-regulated receptor tyrosine kinase pairs with a pseudokinase to activate immune defense upon oomycete recognition in Caenorhabditis elegans
Source: Proc Natl Acad Sci U S A. 2023 Sep 19;120(39):e2300587120. doi: 10.1073/pnas.2300587120 (PMC10523662; doi:10.1073/pnas.2300587120)
Supplement: Supplementary file 1 — Appendix 01 (PDF) [file pnas.2300587120.sapp.pdf]

PNAS

Supporting Information for

A PAX6-regulated receptor tyrosine kinase pairs with a pseudokinase to activate immune defense upon oomycete recognition in *C. elegans*

Florence Drury, Manish Grover, Mark Hintze, Jonathan Saunders, Michael K Fasseas, Charis Constantinou, and Michalis Barkoulas

Corresponding author: Michalis Barkoulas

Email: m.barkoulas@imperial.ac.uk

This PDF file includes:

Figures S1 to S10
Tables S1 to S2
Legends for Datasets S1 to S2

Other supporting materials for this manuscript include the following:

Datasets S1 to S2

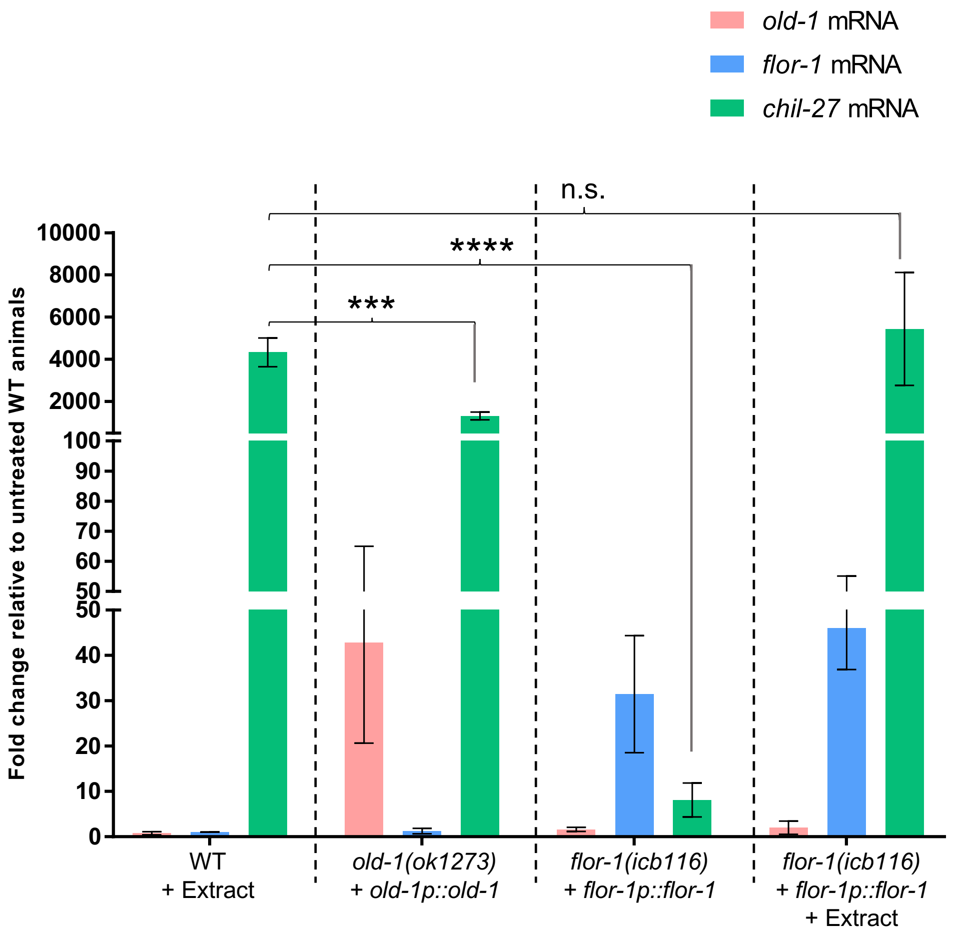

Fig. S1. Levels of *old-1*, *flor-1* and *chil-27* mRNA upon overexpression and extract treatment. RT-qPCR-based quantification of L4 stage animals under all conditions shown in Fig. 1 *C* and *D*. One-way ANOVA and Tukey's multiple comparison test was used to assess statistical significance; p<0.0001****, p<0.001***, p<0.01** and p<0.05*.

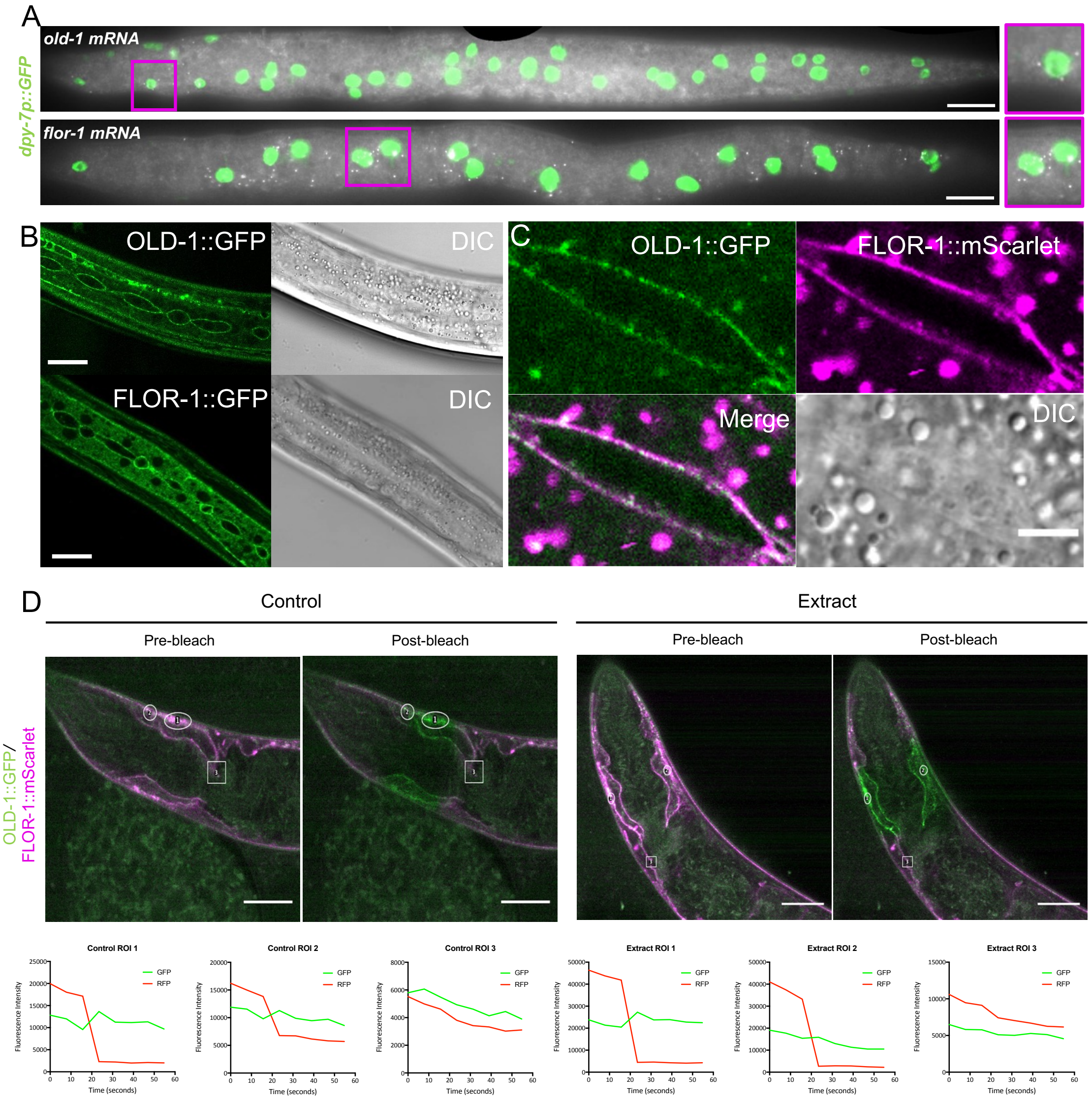

Fig. S2. OLD-1 and FLOR-1 are in close enough proximity to be interacting at the membrane of the epidermis. (*A*) Single epidermal sections of *old-1* and *flor-1* expression by smFISH showing signal surrounding the epidermal nuclei labelled with *dpy-7p::GFP::H2B* (zoomed-in image on the right). Scale bar is 10 μm. (B) Confocal images of OLD-1::GFP and FLOR-1::GFP expression in an apical view of the lateral syncytium. Scale bar is 20 μm. (*C*) OLD-1::GFP and FLOR-1::mScarlet (*icbEx364*) co-localise in the anterior epidermis. Close-up of an anterior seam cell, scale bar is 5 μm. (*D*) Pre-bleach image shows co-localisation of OLD-1::GFP and FLOR-1::mScarlet in the anterior hypodermis. Yellow circle shows regions of interest (ROIs) targeted for photobleaching; yellow square shows control region outside of photobleached zone. Post-bleach image shows fluorescence expression after mScarlet has been photo bleached. Graphs show change in GFP

and mScarlet fluorescence intensity before and after photobleaching. An increase in GFP after mScarlet is bleached indicates that OLD-1::GFP and FLOR-1::mScarlet are within 10 nm of each other on the membrane and possibly interact. Note an increase in GFP expression after mScarlet is bleached both in the presence and absence of extract treatment. This increase is not seen in the negative control (ROI 3). Representative image and graphs, n > 10 per treatment, scale bar is 10 μm.

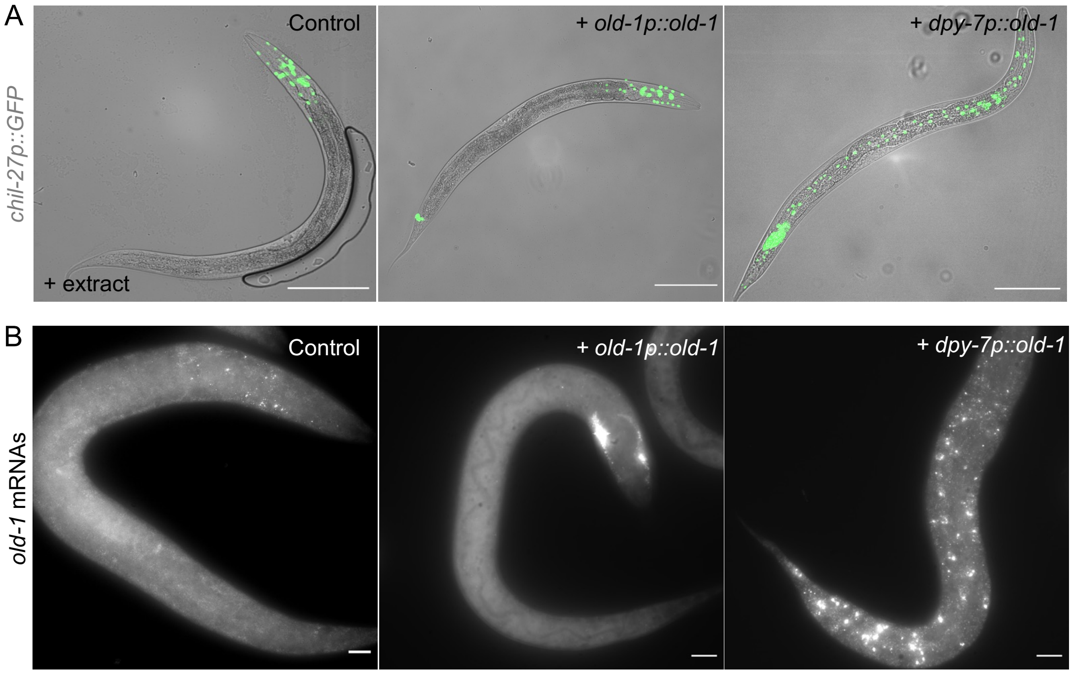

Fig. S3. *dpy-7p::old-1* overexpression causes expansion of *old-1* mRNA distribution throughout the epidermis. (*A*) The *chil-27p::GFP* transcriptional reporter is expressed in an anterior biased gradient upon extract treatment and *old-1p::old-1* overexpression (*icbIs22).* This signal expands throughout the epidermis in *dpy-7p::old-1 (icbEx309)* animals. Scale bar is 100 µm, n > 100, bright signal in the tail is due to the co-injection marker *bus-1p::GFP*. (*B*) In wild type animals, *old-1* mRNAs are expressed in the anterior at low levels. *old-1p::old-1* overexpression (*icbEx358*) leads to increased mRNA number in the anterior epidermis. *dpy-7p::old-1* overexpression (*icbEx359*) results in *old-1* mRNAs being present throughout the body. Scale bar is 10 µm, n > 15.

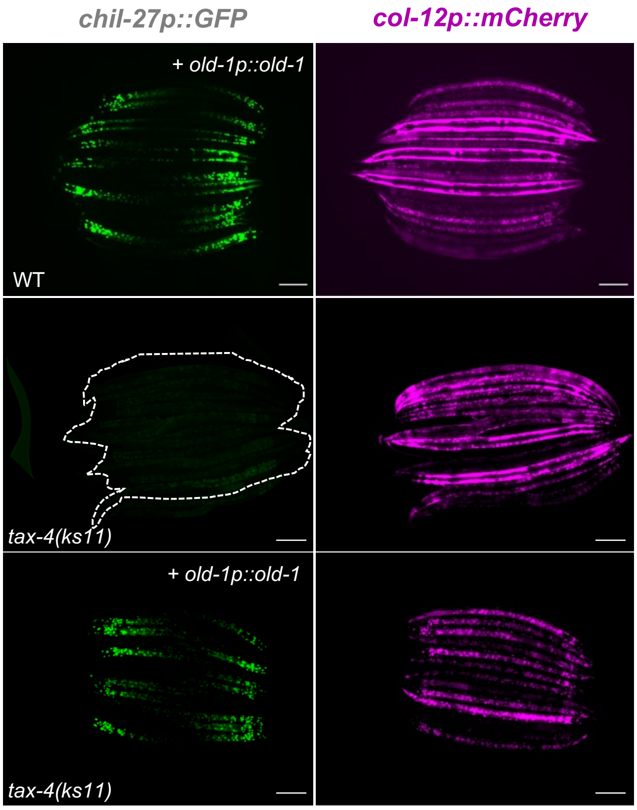

Fig. S4. ***old-1* overexpression does not require TAX-4-dependent signalling for induction of *chil-27p::GFP*.** Loss of *tax-4* function does not suppress the constitutive activation of *chil-27p::GFP* upon *old-1* overexpression (*icbEx424*), n > 100 animals, scale bar is 100 µm, presence of the *chil-27p::GFP* reporter is indicated by *col-12p::mCherry* also present in the same transgene.

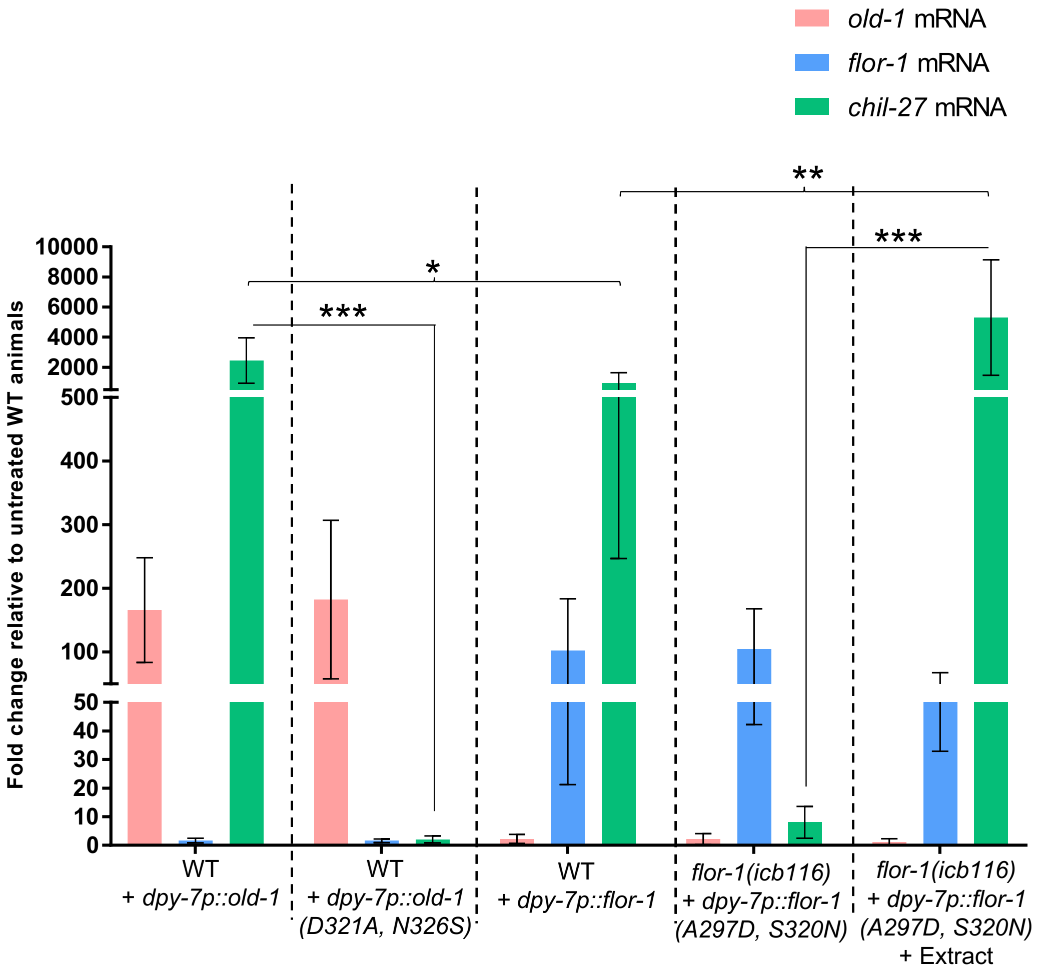

Fig. S5. Levels of *old-1*, *flor-1* and *chil-27* mRNA in animals epidermally overexpressing WT and mutated forms of *old-1* and *flor-1*. RT-qPCR based quantification of L4 stage animals under all conditions shown in Fig. 3 *B* and *C*. One-way ANOVA and Tukey's multiple comparison test was used to assess statistical significance; p<0.0001****, p<0.001***, p<0.01** and p<0.05*.

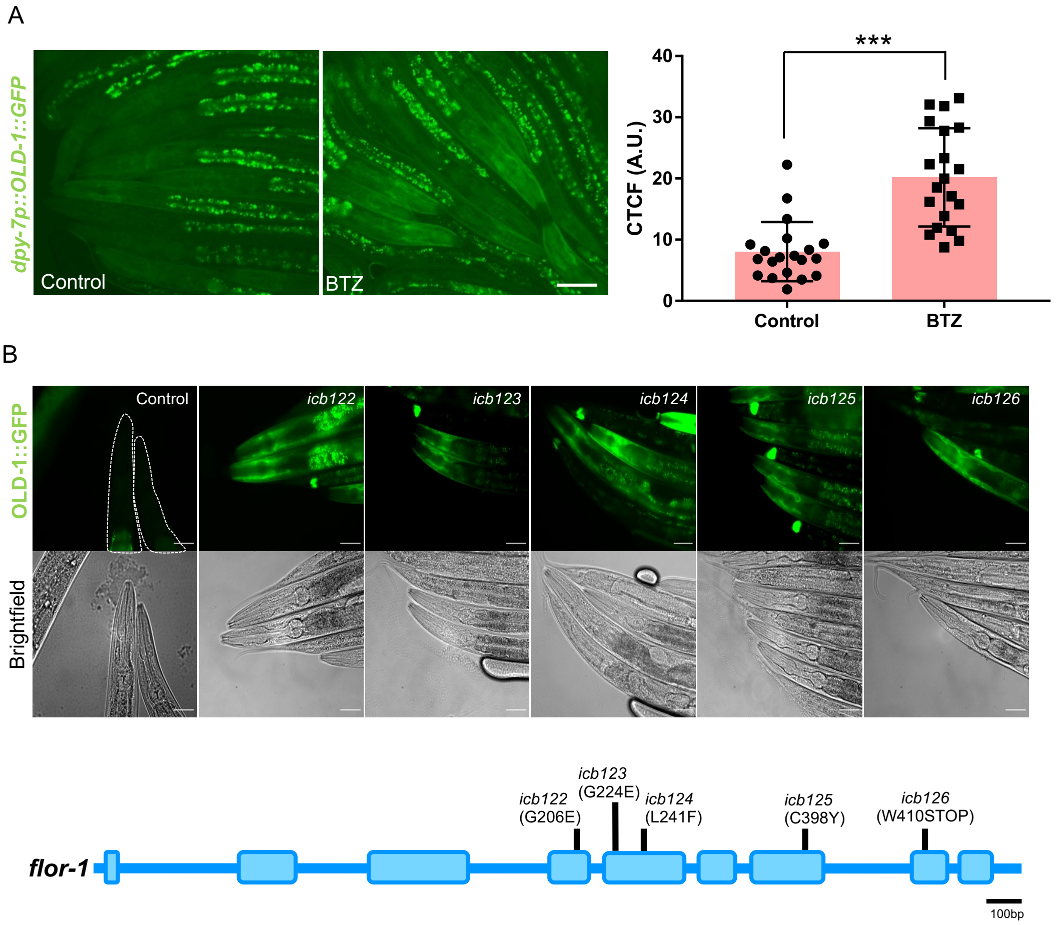

Fig. S6. Inhibition of the proteasome and loss of function of *flor-1* causes OLD-1 accumulation. (*A*) Animals carrying *dpy-7p::OLD-1::GFP* when treated with 20 µM of Bortezomib (BTZ) for 24 hours at L2 stage, show increase in GFP expression (left), suggesting that OLD-1 is kept tightly regulated by the proteasome. Scale bar is 50 µm. The GFP signal in the head region (shown by arrows) of 20 animals under control and BTZ-treated conditions was quantified using FIJI and Corrected Total Cell Fluorescence (A.U.) was plotted (right). Unpaired T-test was used to assess statistical significance, *p*<0.001***. (*B*) Five additional *flor-1* alleles identified in an EMS suppressor screen all showed an accumulation of OLD-1::GFP in the epidermis, indicative of negative regulation of OLD-1 levels by FLOR-1. Bright signal in the tail region corresponds to the co-injection marker *bus-1p::GFP*. Scale bar is 50 µm.

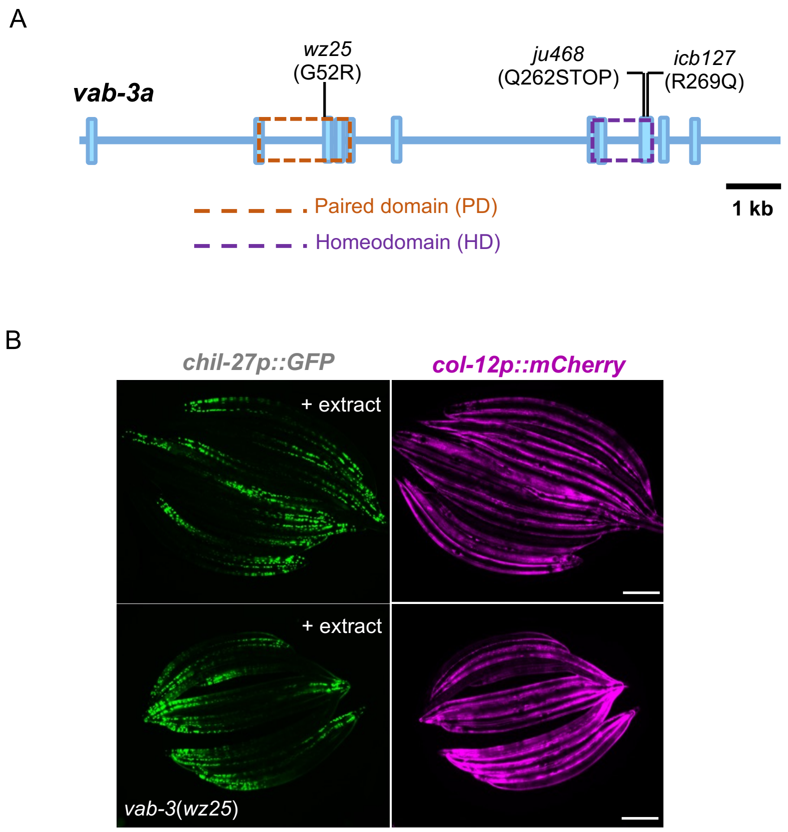

Fig. S7. VAB-3 regulates activation of ORR through its homeodomain (HD). (*A*) Gene structure of *vab-3a* isoform showing location of mutations used in this study. (*B*) *vab-3(wz25)* mutant animals show induction of *chil-27p::GFP* upon extract treatment (n >100 animals and scale bar is 100 μm, presence of the *chil-27p::GFP* reporter is indicated by *col-12p::mCherry* also present in the same transgene).

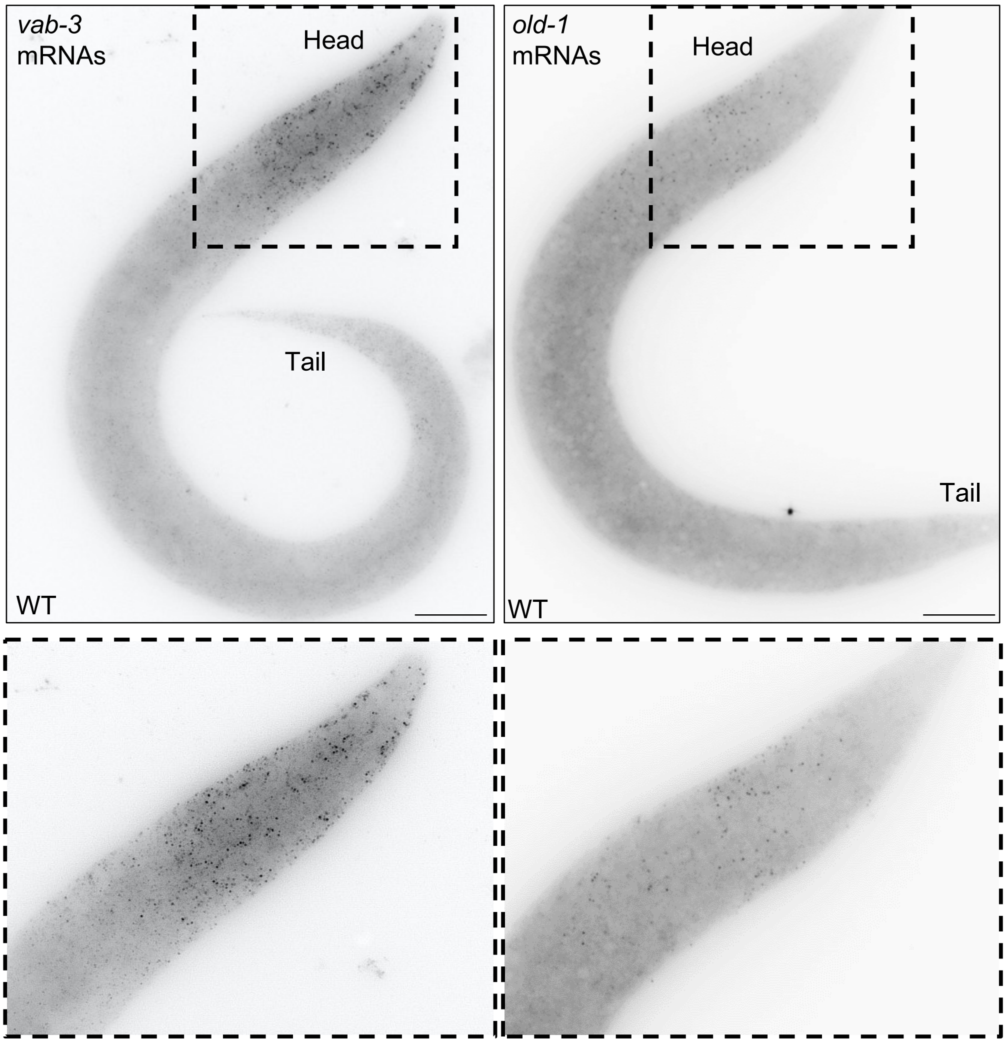

Fig. S8. **Expression pattern of *vab-3* in *C. elegans*.** L1 stage animal showing anterior localization of *vab-3* mRNAs by smFISH, which is reminiscent of *old-1* expression. Scale bar is 10 µm.

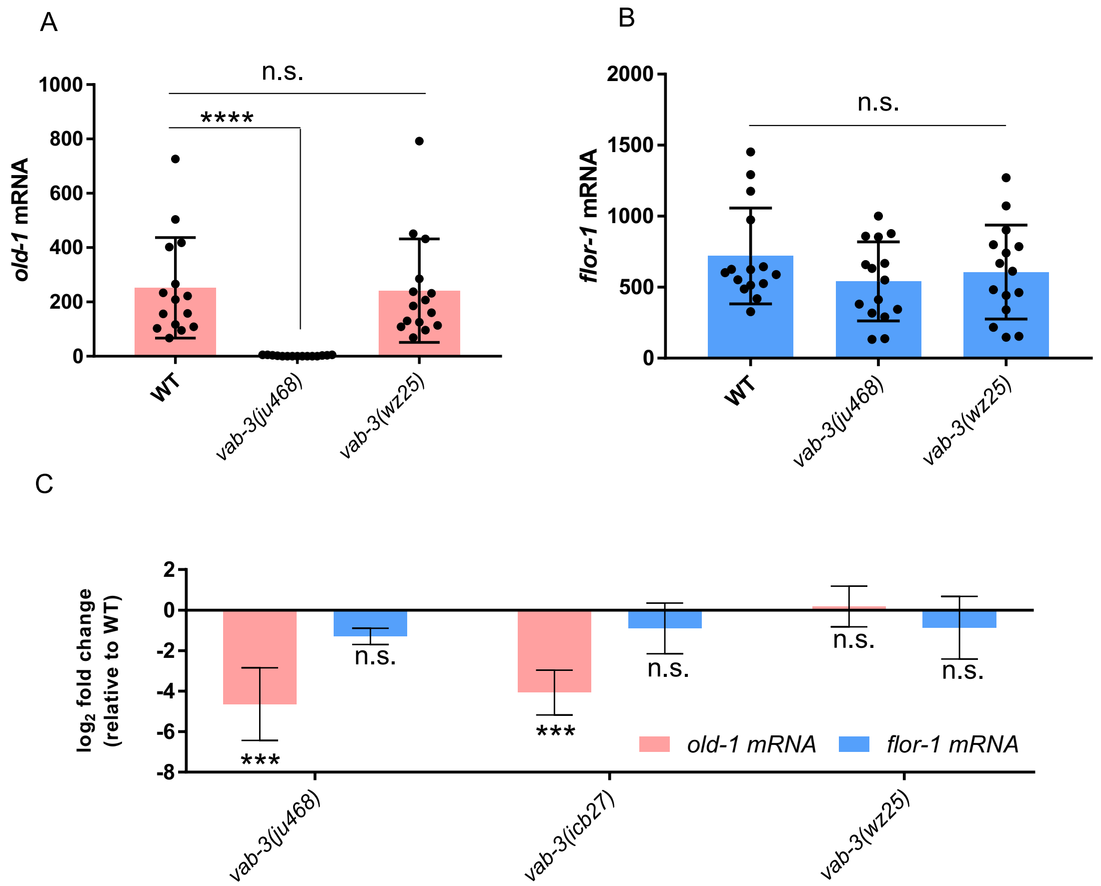

Fig. S9. **Levels of *old-1* and *flor-1* in WT animals and *vab-3* mutants.** smFISH counts of *old-1* (*A*) and *flor-1* (*B*) mRNA at L2 stage in WT, *vab-3(ju468)* and *vab-3(wz25)* mutant animals (n=15, *p*<0.0001*** based on t-test). (*C*) RT-qPCR based quantification of L4 stage WT, *vab-3(ju468), vab-3(icb127)* and *vab-3(wz25)* mutant animals. One-way ANOVA and Tukey's multiple comparison test was used to assess statistical significance; *p*<0.001***.

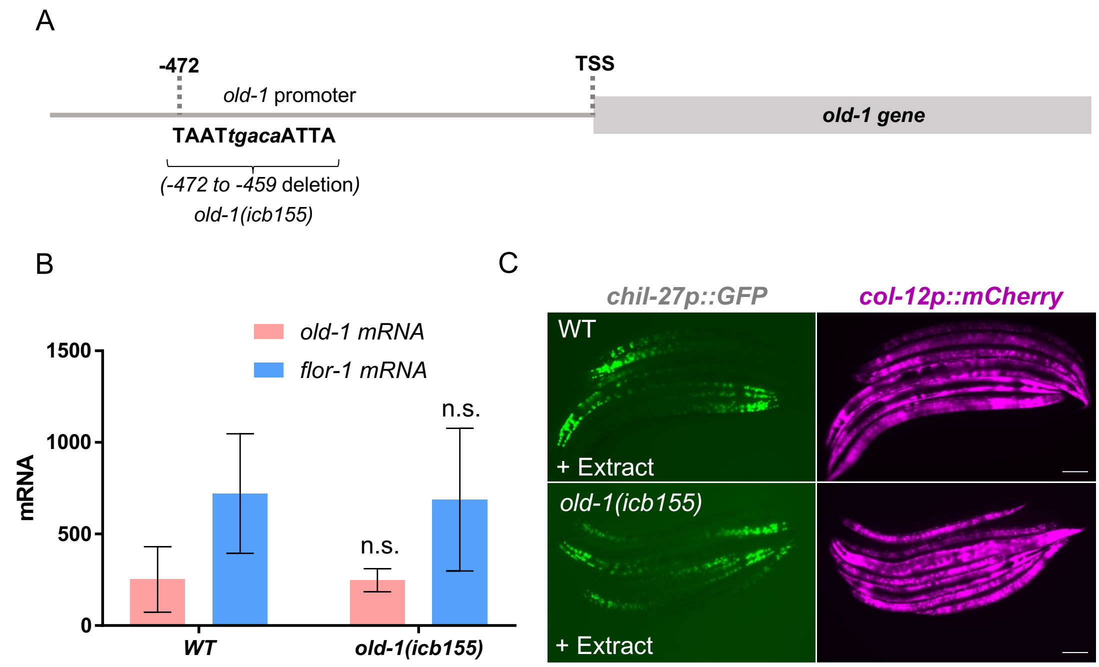

Fig. S10. VAB-3 does not directly regulate *old-1* gene expression through the canonical HD binding site. (*A*) *old-1* gene promoter indicating the homeodomain binding site at position -472 deleted by CRISPR (*icb155* allele). (*B*) smFISH counts of *old-1* and *flor-1* mRNA at L2 stage in *old-1(icb155)* animals compared with WT animals (n=15, n.s. based on t-test). (*C*) *chil-27p::GFP* induction upon extract treatment in *old-1(icb155)* (n > 100 animals and scale bar is 100 μm).

Table S1. Strains used in the study.

Experimental Models: Organisms/Strains	Source	Identifier
M. humicola : Lisbon isolate	Osman et al., 2018	JUo1
C. elegans: wild-type N2	Caenorhabditis Genetics Center	N2
C. elegans: icbIs4[chil-27p::GFP, col-12p::mCherry-pest] II	Osman et al., 2018	MBA281
C. elegans: icbIs5[chil-27p::GFP, col-12p::mCherry-pest] IV	Osman et al., 2018	MBA282
C. elegans: tax-4(ks11) III; icbIs5[chil-27p::GFP, col-12p::mCherry-pest] IV	Fasseas et al., 2021	MBA668
C. elegans: old-1(icb118)II;icbIs5[chil-27p::GFP, col-12p::mCherry-pest] IV	This study	MBA1038
C. elegans: old-1(icb119)II;icbIs5[chil-27p::GFP, col-12p::mCherry-pest] IV	This study	MBA1037
C. elegans: old-1(icb117)II;icbIs5[chil-27p::GFP, col-12p::mCherry-pest] IV	This study	MBA418
C. elegans: old-1(icb133)II;icbIs5[chil-27p::GFP, col-12p::mCherry-pest] IV	This study	MBA1032
C. elegans: old-1(ok1273)II	Caenorhabditis Genetics Center	RB1215
C. elegans: old-1(ok1273)II; icbIs5[chil-27p::GFP, col-12p::mCherry-pest] IV	This study	MBA1018
C. elegans: flor-1(icb93)V; icbIs5[chil-27p::GFP, col-12p::mCherry-pest] IV	This study	MBA517
C. elegans: flor-1(icb134)V;icbIs5[chil-27p::GFP, col-12p::mCherry-pest] IV	This study	MBA1035
C. elegans: flor-1(icb120)V;icbIs5[chil-27p::GFP, col-12p::mCherry-pest] IV	This study	MAB1033
C. elegans: flor-1(icb116)V;icbIs5[chil-27p::GFP, col-12p::mCherry-pest] IV	This study	MBA1415
C. elegans:icbEx308[old-1p::OLD-1::GFP; bus-1p::GFP,pBJ36]; icbIs4[pGO4,pCMH1195]IV	This study	MBA1013
C. elegans: icbEx435[pER6 (flor-1p::flor-1::Scarlet), pIP1303F(rps-0::hgb:unc-54), pBJ36], flor-1(icb116); icbIs5[pGO4, pCMH1195] IV	This study	MBA1447
C. elegans: flor-1(icb116)V	This study	MBA1217
C. elegans: icbEx360[PIP1303F(rps-0::hgb:unc-54), pBJ36]	This study	MBA1270
C. elegans: icbEx358[old-1p::old-1, PIP1303F(rps-0::hgb:unc-54), pBJ36]	This study	MBA1271
C .elegans: icbEX359[pFD20(dpy-7::old-1::unc-54), PIP1303F(rps-0::hgb:unc-54), pBJ36]	This study	MBA1272
C. elegans: unc-119(ed3) III; icbSi3[dpy-7::GFP::H2B::unc-54 3'UTR+cb-unc-119]	This study	MBA260
C. elegans: icbEx310[old-1p::OLD-1::GFP, pRF4(rol-6(su1006)),pBJ36]	This study	MBA1089
C. elegans:rde-1(ne219) V;icbIs5[pGO4, pCMH1195] IV xkIs99.	This study	MBA576
C. elegans: icbEx309[pFD20(dpy-7p::old-1::unc-54 3'UTR); bus-1p::GFP]; icbIs5[pGO4, pCMH1195] IV	This study	MBA1088
C. elegans: icbEx316[pFD23(dpy-7p::old-1(D321A, N326S)::GFP), myo2p::RFP, pBJ36], icbIs5[pGO4, pCMH1195]IV	This study	MBA1190
C. elegans: icbEX364[old-1p::old-1::GFP, pER6(flor-1p::flor-1::mScarlet), PIP1303F(rps-0::hgb:unc-54)]	This study	MBA1276
C. elegans: icbEx433[old-1p::old-1, pIP1303F(rps-0::hgb:unc-54), pBJ36]; flor-1(icb116); icbIs5[pGO4, pCMH1195] IV	This study	MBA1441
C. elegans:icbEx434[pFD20(dpy-7p::old-1), pIP1303F(rps-0::hgb:unc-54), pBJ36],flor-1(icb116); icbIs5[pGO4, pCMH1195] IV	This study	MBA1442
C. elegans: icbEx427[pJS2(dpy-7p::flor-1), PIP1303F(rps-0::hgb:unc-54), pBJ36], old-1(ok1273), icbIs5[pGO4, pCMH1195] IV	This study	MBA1460
C. elegans: icbEx429[pFD21(dpy-7p::old-1::GFP), PIP1303F(rps-0::hgb:unc-54), pBJ36]	This study	MBA1462
C. elegans: icbEx436[pFD21[dpy-7p::old-1::GFP], PIP1303F(rps-0::hgb:unc-54), pBJ36], flor-1(icb116); icbIs5[pGO4, pCMH1195] IV	This study	MBA1448
C. elegans: icbEx414(old-1p::old-1::GFP, pIP1303F(rps-0::hgb:unc-54), pBJ36]; flor-1(icb116); icbIs5[pGO4, pCMH1195] IV	This study	MBA1414
C. elegans: flor-1(icb122); icbIs22(old-1p::old-1::GFP, bus-1p::GFP, pBJ36); icbIs5[pGO4, pCMH1195] IV	This study	MBA1335
C. elegans: flor-1(icb123), icbIs22(old-1p::old-1::GFP, bus-1p::GFP, pBJ36); icbIs5[pGO4, pCMH1195] IV	This study	MBA1338
C. elegans: flor-1(icb124), icbIs22(old-1p::old-1::GFP, bus-1p::GFP, pBJ36); icbIs5[pGO4, pCMH1195] IV	This study	MBA1337
C. elegans: flor-1(icb125), icbIs22(old-1p::old-1::GFP, bus-1p::GFP, pBJ36); icbIs5[pGO4, pCMH1195] IV	This study	MBA1334
C. elegans: flor-1(icb126), icbIs22(old-1p::old-1::GFP, bus-1p::GFP, pBJ36); icbIs5[pGO4, pCMH1195] IV	This study	MBA1342
C. elegans: vab-3(icb127), icbIs22(old-1p::old-1::GFP, bus-1p::GFP, pBJ36); icbIs5[pGO4, pCMH1195] IV	This study	MBA1339
C. elegans: icbEx437[WRM0640cC05, myo-2::dsRed, BJ36];vab-3(icb127);icbIs22(old-1p::old-1::GFP; bus-1p::GFP); icbIs5[pGO4, pCMH1195] IV	This study	MBA1433
C. elegans: icbEx438[pFD20(dpy-7p::old-1), myo-2::dsRed, pBJ36]; vab-3(icb127);icbIs22(old-1p::old-1::GFP; bus-1p::GFP); icbIs5[pGO4, pCMH1195] IV	This study	MBA1436
C. elegans: vab-3(ju468) X	Caenorhabditis Genetics Center	RB693
C. elegans: icbEx452[pJS2(dpy-7p::flor-1), PIP1303F(rps-0::hgb:unc-54), pBJ36]; icbIs5[pGO4, pCMH1195] IV	This study	MBA1449
C. elegans: icbEx424[old-1p::old-1, pIP1303F(rps-0::hgb:unc-54), pBJ36];tax-4(ks11); icbIs5[pGO4, pCMH1195] IV	This study	MBA1457
C.elegans: icbEx356[pFD25(dpy-7::old-1::mCherry::unc-54),flor-1p::flor-1::GFP, PIP1303F(rps-0::hgb:unc-54)]	This study	MBA1210
C. elegans: icbIs22(old-1p::old-1::GFP, bus-1::GFP, BJ36);ju468 (vab-3); wIs51[scm::gfp + unc-119+] V; icbIs4[pGO4, pCMH1195] II	This study	MBA1435
C. elegans: vab-3(ju468)X; wIs51[scm::gfp + unc-119+] V; icbIs4[pGO4, pCMH1195] II	This study	MBA1434
C.elegans: flor-1(icb136[flor-1::gfp])	This study	MBA998
C. elegans: icbEx426[pJS4(dpy-7p::flor-1(A297D, S302N)::GFP, pIP1303F(rps-0::hgb:unc-54)], flor-1(icb116); icbIs5[pGO4, pCMH1195] IV	This study	MBA1459
C. elegans: icbEx493[pJS7(dpy-7p::flor-1(A297D, S302N, Y420F, Y452F)::GFP, pIP1303F(rps-0::hgb:unc-54), pBJ36], flor-1(icb116); icbIs5[pGO4, pCMH1195	This study	MBA1535
C. elegans: icbEx501[pJS6(dpy-7p::flor-1(A297D, S302N, Y327F, Y328F)::GFP, pIP1303F(rps-0::hgb:unc-54), pBJ36], flor-1(icb116); icbIs5[pGO4, pCMH1195	This study	MBA1558
C. elegans: ynIs64[Pflp-17::GFP]I; vab-3(wz25)X	Brandt et al., 2019	FQ457
C. elegans: vab-3(wz25)X; icbIs5[pGO4, pCMH1195] IV	This study	MBA1725
C. elegans: old-1(icb155)	This study	MBA1723
C. elegans: old-1(icb155); icbIs5[pGO4, pCMH1195] IV	This study	MBA1724

Table S2. Oligos used in the study

Oligo	Sequence	*old-1 s* mFISH probe	*flor-1* smFISH probe	*vab-3* smFISH probe
old-1FullF	GGAACAGCGAGGAGAGGAAA	gtattgcagtgagcaaatcc	acagtaatgacgtcatgaca	agttgattcacaccagtgtg
old-1FullR	TCGGCGTCTTTCCAAAATGT	atcttcaaagtttcgactca	gcttgttcggttaatccaat	gtggacgcccattaacaaat
T01G5.1promF	AAAACGACGGCCAGTGAATTCATTTAAATCTGAACTATAGGAATCGGCCGT	ccagtttcatctttatcagt	tgatcttagcagtctctcgg	gattctttgacgtgttgcat
T01G5.1FullR2	CTGCACAGTCAACGTCATGT	cttcctgtacattcgaatct	aatcatttgagccgtctttc	catcctttgtgcgctaaatc
dpy-7old-1gibsonfwd	TCTTACATTTTGTTCCAGATAAGTTTAAACATGAAAGGCACCTTAATTTT	gagttctcgtttccttgaat	gccaaaaacatcgatttcca	cccgttggaaacctgtaata
dpy-7old-1gibsonrev	TTGGACTTAGAAGTCAGAGGCAATTTAAATTTAACTATCACAATGTTTCT	tttcttcttttatgatccgc	aaggagtatgcatccaagtg	ggcacaagattttggagaca
old-1kinasemutF	TGT GTT CAT CGT GCT TTG GCT CTC CGC AGT GTA CTG ATA AAG AA	ctttctccggatgtaattgg	ggtatccgataaatagtagc	attgtgcctgactcgtagta
old-1kinasemutR	TTC TTT ATC AGT ACA CTG CGG AGA GCC AAA GCA CGA TGA ACA CA	ctctggaggacattctacat	tctcagcttagatatcttcc	tggttttgagccaccaattg
T01G5.1R_pER2/4	TTGATAACTGCCTCTCCCTTGCTGACCATCGACATGAAATTGTATCTGAAAGC	ttttttcgttaattggggct	cccagtttgtctttcaattt	attttctcgacgacgtcact
dpy-7T01G5.1gibF	ACATTTTGTTCCAGATAAGTTTAAACATTTATGACGTCATTACTGTTCATATTCCTTTTG	gatcttcctcattgatttca	actccaaatgagtttgtgca	ggttggtcacgtttgtaatc
T01G5.1unc-54 gibR	TTGGACTTAGAAGTCAGAGGCAATTTTTACTTGTAGAGCTCGTCCATTCC	tagtggtcttccctttgaaa	cagcggagtagattcttcag	ctctgatttcccatgcaaaa
GFPoATG-F	ATGAGTAAAGGAGAAGAATTGTTCACTG	tcgtattattccaaactccc	gttttttggttattaaccgg	gcagatattatcagctagca
GFPoSTOP-R	TTACTTGTAGAGCTCGTCCATTCC	gcacacatcactttcaattc	gacttggatccaattgtact	tacgcttggtattgtttcgt
T01G5.1Frep	attttggagaaaatgttaatacaaacatttcagATTGGAAAACAAATTACGGAGAAGTTAAAATCTGCTAAAAATTACCAGGACAAGTTAAAGATTGGAACGTCTGAAAATCGTTTTTTAGGAGCTTCTGGTGCATCAGGAGCATCTATGAGTAAAGGAGAAGAATTGTTCACTG	aatgtttgggtgtttaccga	atttccttttcttcgatgat	ttccgtagaaccctatttat
T01G5.1Rrep	attttggagaaaatgttaatacaaacatttcagATTGGAAAACAAATTACGGAGAAGTTAAAATCTGCTAAAAATTACCAGGACAAGTTAAAGATTGGAACGTCTGAAAATCGTTTTTTAGGAGCTTCTGGTGCATCAGGAGCATCTATGAGTAAAGGAGAAGAATTGTTCACTG	gtaattcctccgactaaagc	tatcctgctgtaatagtgtt	tgtgacttgctcctttttttg
T01G5.1fwd	GAGAATTTGGACTCGCACGG	ctaagaagatccccgttctc	catagatttcatctttccca	tatcgtaaagctctgtttgc
dpy-7 T01G5.1 gib F	ACATTTTGTTCCAGATAAGTTTAAACATTTATGACGTCATTACTGTTCATATTCCTTTTG	tattcgattgtctctcaaga	tcggtgaatttttatgcgct	ccataccaactggaattgta
T01G5.1 unc-54 gib R	TTGGACTTAGAAGTCAGAGGCAATTTTTACTTGTAGAGCTCGTCCATTCC	gtccattgatcgtttgtgaa	ggatcattttctgatgggtt	attcatcggtattggccatt
T01G5.1 SDM F	GGTGCCTTCATCGTGATCTGGCATTGAGAAATATATTTGTGACACG	agaatcttgttcagtttcta	gaaagtgttaccagctcatc	atgggtttaatccaactgct
T01G5.1 SDM R	CGTGTCACAAATATATTTCTCAATGCCAGATCACGATGAAGGCACC	tgagagcaagtcaacgaggc	gccaatatgcaggaatgtga	agtctttggttcgatgagtg
FLOR-1 SDM AL F2	ATGCTAGAAAAGAATTTTTCATGTAAGCAGGTCG	caaatactccatccctttttg	ttctcgaacatatcccacta	catctttctcaaactctcct
FLOR-1 SDM AL R2	CGACCTGCTTACATGAAAAATTCTTTTCTAGCAT	ctttatcagtacattgcgga	ccgatacgatcagtagcata	tcagttggaggtttttggtc
T01G5.1 Y420 SDM F	AAGCCCGTCCCACCTTCTCTAAGTGTGTCGA	gccaatccaaagtctgcaat	attgatcaagtcttccgtgc	aacttccgtttcaatcgcat
T01G5.1 Y420 SDM R	TCGACACACTTAGAGAAGGTGGGACGGGCTT	atctttatttttatgcctcc	agttcagtttgcgatcgatc	gcgtaaacgaggttctgttt
T01G5.1 Y452 SDM F	AATCTGCTAAAAATTTTCAAGACAAGCTGAA	ccagtgtattggaagcggtg	cggaacaagtgagttggtta	tcaagactctcaatctggac
T01G5.1 Y452 SDM R	TTCAGCTTGTCTTGAAAATTTTTAGCAGATT	ttgtctatactttcaggtgc	taaactttctggttactgcc	ctggataatgagtcctttca
RMDW0838	TTTCATTAATTGACAATTAGGTTTTAGAGCTATGCT	ctgatttctgagtgaacagt	tcttgttctccattgttgaa	aagacgctcccttgcaaaaa
old-1 repair	ATTCTGTAGAAAGAAAGATACCGCGATGTTTTTCATAGTGGATGAGTAATGTATCCTTTCCGTTTTGTCTTTTTTCTTCAAAAAATAAAACTAAAACCCTTTAACTTTTTGATCGAACATAACATAAAACATATTTATATTTTGTTGAATATTTGATATTGAAAGTTATTAATACATAGTGAAGTAAATGAAAATTATAT	agttcataaagacagacccc	ctgcatatcatcactaatcc	tcttgcttctggtagttgaa
old-1_CRISP_F	GATACCGCGATGTTTTTCATAGTGG	ttcataaggtgattttccca	gaagatccaatgtgcacagt	cgattcgagaaccatacctg
old-1_CRISP_R	CTTTCATTCCACGCCTCTGG	gtaagacagcacatacttcc	gcaatggcaatttgatatcc	gatgatcgcttgtttctcat
TT01G5.1RNAiF	AGACCGGCAGATCTGATATCATCGATGAATCTACTCCGCTGATTCCG	caagtcttttcccttcattc	ccttgaatcagccaagaact	tggcgttccattggataatg
TT01G5.1RNAiR	TCGACGGTATCGATAAGCTTGATATCGCCGCACTTCAGATTTCTCCG	catatctagatcccagcata	caatgccagagcacgatgaa	tgttacttccagtcactgat
pmp-3_qRTF1	CGGAGGAAAAACTGGTCAAG	ctcatttgatgttgttttca	ttgttttatttcgtgtcaca	ggagatccaatcggatttgt
pmp-3_qRTR1	TGCAACGAGAGCAACTGAAC	gagctttctggtgagatcaa	cgagtccaaattctccgatg	tggaagggaatcgattcggc
chil-27qRT-F	tcaagtggaggactgcaaca	gtcgaagttgattgttggtt	tttatcctcagaactttccg	aggcacaaagtttgttgtcg
chil-27qRT-R	tgagttattttcggtagattccagt	tgtttctcatcagaaagcca	acttcagatttctccgtgaa	cagcatacatctgactcgtt
old-1 qPCR Fwd	ATGTCTCCAGAGCTAGAAT		aaggcatactgcaaacgacc	gaatatggatccattgctgg
old-1 qPCR Rev	TAACGGTAATTTCACTGCCA		gtgggactcctaattggaat	ccattgaaaaaccattggca
T01G5.1 qPCR Fwd	GCCCGAGAGACTGCTAAGAT		cctgaccacaataaacagcg	tctgttacttgtggatatgg
T01G5.1 qPCR Rev	TTGTGCAGAGACCCCAGTTT		attccggaagttttctttca	ggaacatgtgatgtggttgg
			catatctggatggcagtact	aaatcatacggcgatcttcc
			tcagggtcaaagttccaaca	tggtcggcattcttggatat
			acacacttagagtaggtggg	tgattgttggaaaccatgcc
			ttctccgtaatttgttttcc	aaagtgtcggaatgtctcct
				attgagcactgcagaaaccg
				gagtcagagacgggtcaatg
				gctgagttcatgcatttgtg
				gaggtctccaataatgttct

Dataset S1 (separate file). Differentially expressed genes upon extract treatment in N2, *old-1(-)* and *flor-1(-)* animals vs no extract treatments, and in animals overexpressing *old-1 (+ old-1p::old-1)* compared with non-transgenic animals.

Dataset S2 (separate file). Differentially expressed genes upon epidermal overexpression of *old-1 (+ dpy-7p::old-1)* compared with WT animals carrying the co-injection marker (hygromycin resistance).
